# Trajectory analysis and optimization of sea buckthorn fruit vibration separation manipulator based on I-PSO algorithm

**DOI:** 10.1038/s41598-023-47001-2

**Published:** 2023-11-17

**Authors:** Bingqin Liang, Xinzhang Lin, Ganghui Liu, Jin Lei, Weibing Wang

**Affiliations:** College of Mechanical and Electrical Engineering; Xinjiang Production and Construction Corps Key Laboratory of Modern Agricultural Machinery; Key Laboratory of Northwest Agricultural Equipment, Ministry of Agriculture and Rural Affairs, Shihezi University, Shihezi, 832003 China

**Keywords:** Mechanical engineering, Software

## Abstract

In this paper, the optimal time planning of vibration separation trajectory of Hippophae rhamnoides fruit is studied for space manipulator using the I-PSO algorithm. The first step is to analyze the motion of the robotic arm's joints, which are limited in range and speed, in combination with a 3–5–3 polynomial interpolation, an improved Particle swarm optimization with adaptive inertia weight and asynchronous learning factor is proposed, and the specific process is given. Experimental images and data show that the improved particle swarm optimization algorithm can ensure the continuity of joint acceleration and velocity, and the optimal vibration trajectory time is 0.536539094 s Compared with the planned system trajectory time of 0.71022 s, the speed increased by 24.5%. The results of the orthogonal experiment show that the average fruit drop rate reaches 96.19%, which verifies the validity and reliability of the I-PSO algorithm for optimal time planning of seabuckthorn fruit separation vibration trajectory.

## Introduction

Sea buckthorn (Hippophae rhamnoides L.), also known as vinegar willow, blackthorn; elaeagnaceae sea buckthorn, is known as the 'shining horse' by Greece^1–3^. The root of sea buckthorn is resistant to cold, drought, and barrenness due to nitrogen fixation by rhizobia, a high-quality ecological tree species for improving soil and the ecological environment^4–6^. Due to its strong attachment, mature fruit can be crushed by small external forces, resulting in very difficult harvesting. Mechanical harvesting has become a bottleneck problem restricting the large-scale industrial production of the sea buckthorn industry^7^. Among them, the vibration trajectory analysis and optimization methods of the manipulator are very important to realize the mechanization of vibration separation of sea buckthorn fruit.

Time trajectory planning of a manipulator is a process of determining the trajectory of a manipulator under given tasks and constraints. It is very important to ensure safety, improve efficiency, improve precision, adapt to dynamic environments and realize coordinated motion. The time trajectory planning of manipulators is related to the limitation of time efficiency of enterprises and is also an important research point in robot kinematics^8–11^. Hector et al. simulated and predicted the vibration behavior of fruit by finite element analysis, which provided a theoretical basis for further improving fruit sorting equipment and technology^12^. Torregrosa et al. used artificial vision techniques to record the movement of fruits during vibration using a camera under the condition of applying vibration, and extracted the separation effect of citrus fruits by image processing and analysis techniques^13^. To optimize the trajectory of the manipulator, Yong et al. put forward a genetic algorithm optimization method based on a transition rectangle^14^. Yan Li et al. applied the coordinated motion characteristics of biology to the motion planning of a manipulator in two-dimensional space and proposed a new biological method to plan the motion of an artificial manipulator^15^. Hao Tian et al. proposed a neural network-based trajectory planning method for robotic manipulators and verified the feasibility and effectiveness of the method through several experiments^16^. The above research has made a great contribution to the research of trajectory planning and optimization of manipulator. At the same time, we can see that the multi-objective optimization method is not perfect, and the traditional particle swarm optimization algorithm is easy to fall into the local optimization, resulting in slow convergence speed, local optimization imbalance and so on. Therefore, in order to solve the problem that traditional algorithms can easily fall into the local optimum, this study chooses to analyze the branch picking and the vibration separation trajectory of seabuckthorn, and optimizes the trajectory time through the improved PSO algorithm. It provides an experimental basis for studying the vibration separation trajectory of sea buckthorn fruit, improves the working efficiency and reliability of the manipulator used for vibration separation of sea buckthorn fruit, improves the production environment, and promotes the development and progress of robot technology.

## Materials and methods

### Materials

Seabuckthorn comes from the Xinjiang production and Construction Corps 9th Division 170th Regiment Seabuckthorn planting base. The variety is the “Late autumn red” variety whose tree age is about 4 years. The plant-row spacing is about 2–4 m, the plant height is about 2–2.5 m, the width is about 2 m.

### Methods

#### Trajectory analysis

##### Select the trajectory planning method

Trajectory planning is divided into joint space trajectories and task space trajectories. Angle-space trajectory: first, the angle of two time points in ${t}_{0}\sim {t}_{1}$ is interpolated to ensure the continuity of the angle, and then the inverse kinematics trajectory planning of the angle interpolation. Task space trajectory: firstly, interpolate the position displacement from ${t}_{0}\sim {t}_{1}$ to ensure the consistency of the displacement, then plan the inverse kinematics trajectory of the position in space.

Since the robot arm can determine the position of the mission space at an unknown joint angle, this research chooses the mission space method for trajectory planning.

##### Pick up trajectory planning

The JTRAJ function generates robot trajectories quickly and smoothly. The continuous polynomial trajectories generated by the Trajectory of joint space (JTRAJ) function can realize the drop of branches and leaves in the picking process. The joint position at the time point was obtained by entering the starting position, the ending position, and the time step, and the planned trajectory was obtained^17^. This trajectory ensures continuity of joint acceleration and velocity, more in line with actual robot motion.

**Figure 1:**
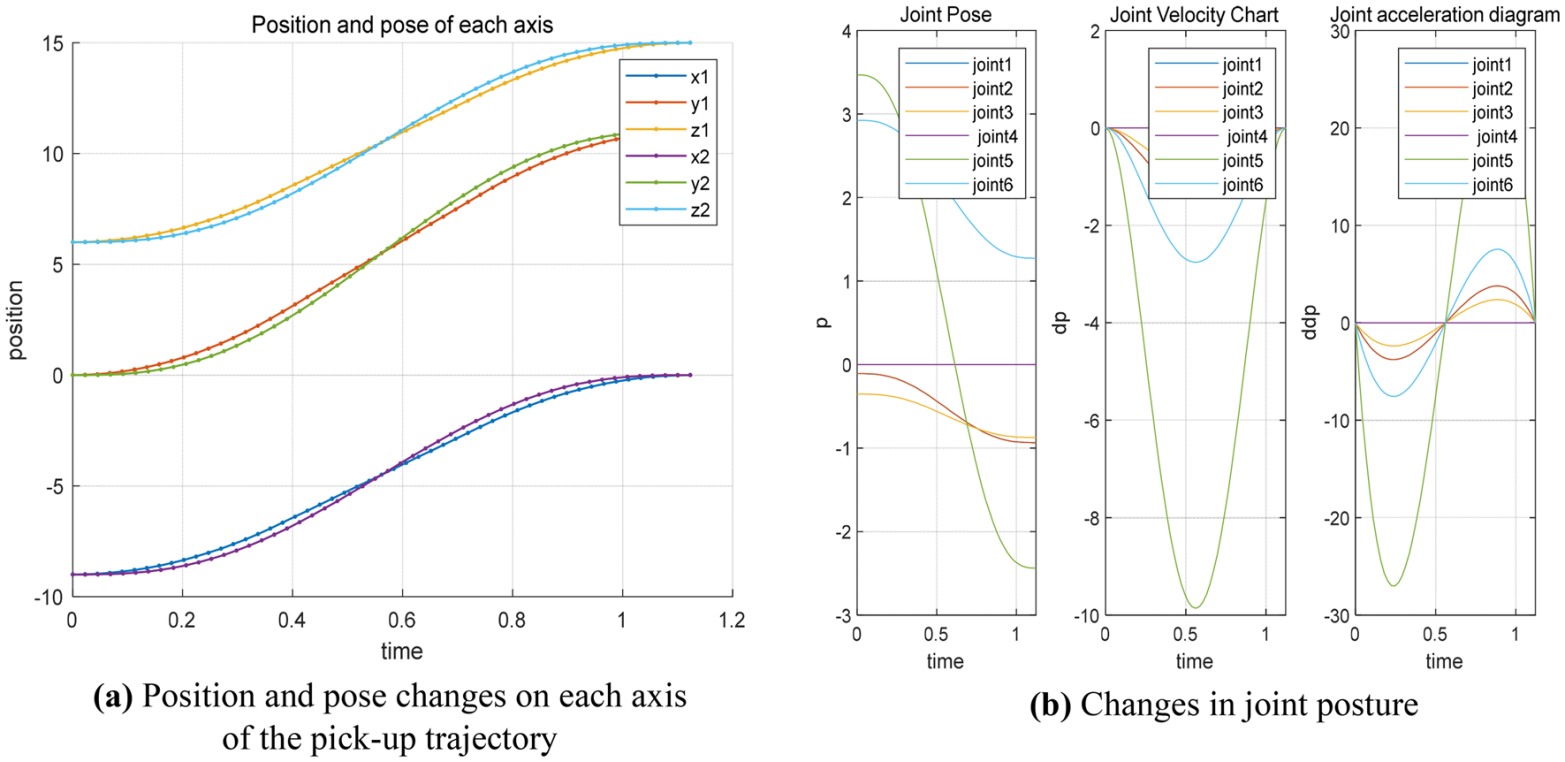
JTRAJ function is used to pick up the parameters of the robot arm.

It can be found from Fig. 1. that the JTRAJ function is smooth when planning the pick-up trajectory, and there is a slight vibration of the trajectory, which is beneficial to the drop of branches and leaves during the pick-up process.

##### Vibration trajectory planning

The path planning of seabuckthorn fruit vibration separation is to make the system vibrate according to the predetermined path or path through reasonable design and control.

**Figure 2:**
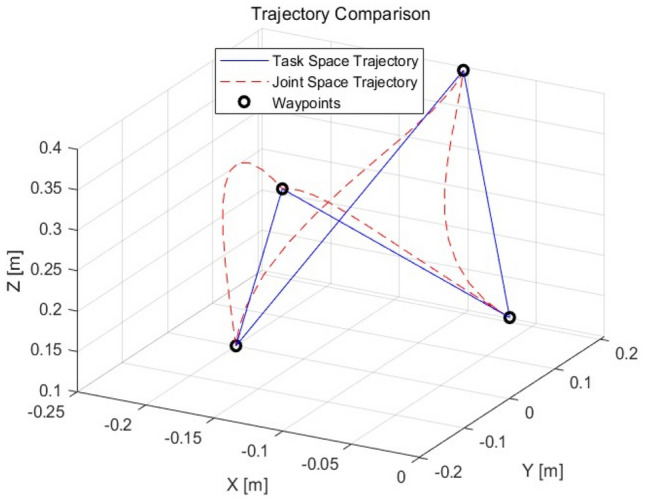
Trajectory of joint angle and task space angle.

The joint space and mission space trajectory planning points for the seabuckthorn fruit vibration separation manipulator were set to (0, 0, 0.2), (− 0.1, 0.2, 0.4), (− 0.2, 0, 0.1), (− 0.1, − 0.2, 0.4), (− 0.1, − 0.2, 0.4), (− 0.1, − 0.2, 0.4), (− 0.1, − 0.2, 0.4), the last loop returns at the initial position (0,0,0.2). The simulation results are shown in Fig. 2.

**Figure 3:**
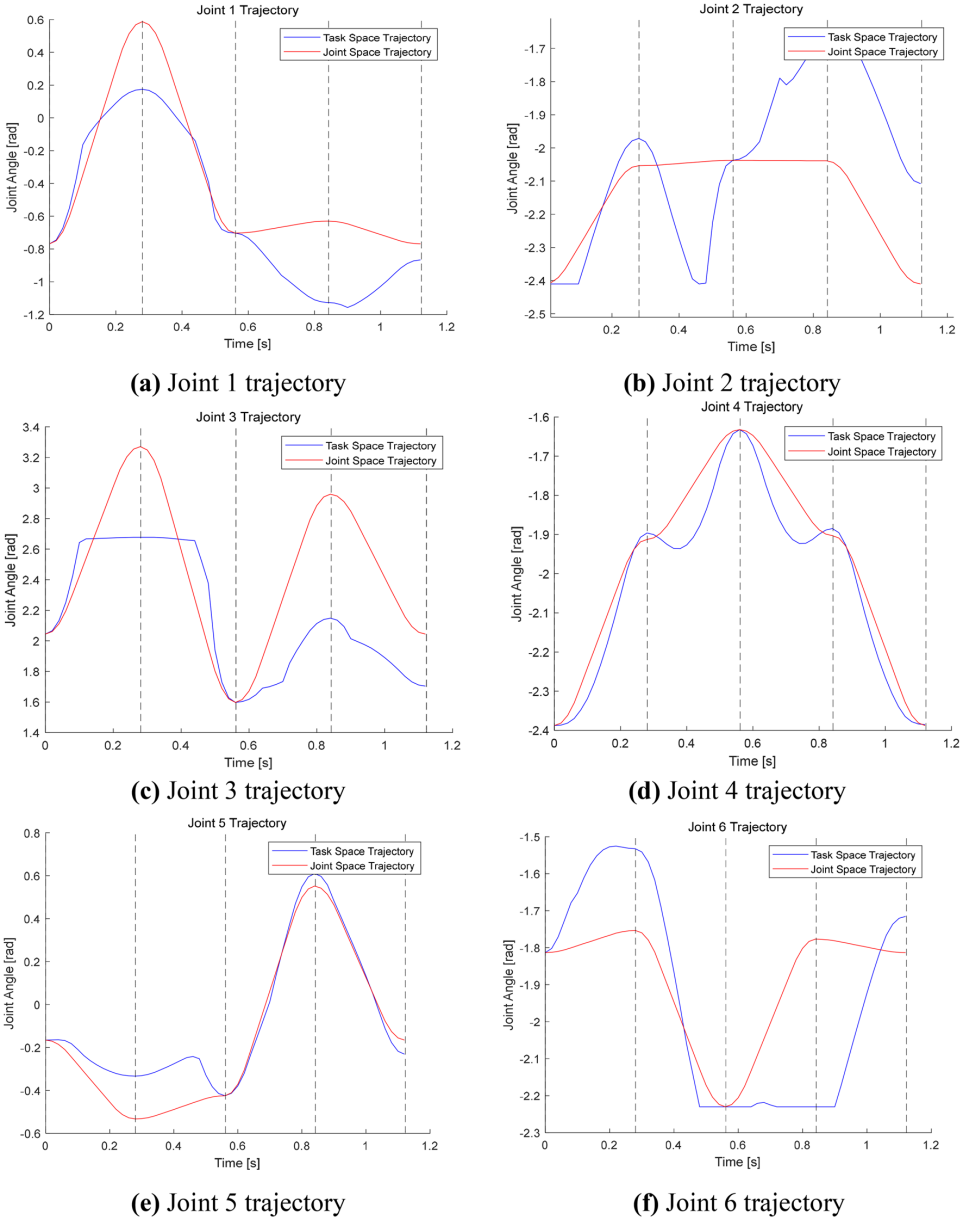
The vibration track of each joint of the manipulator.

**Figure 4:**
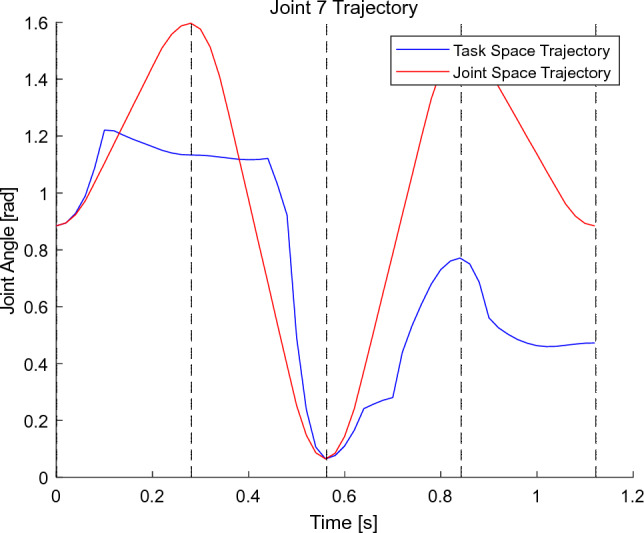
The end of the manipulator clamps the track of the component.

As shown in Fig. 3, the space trajectory time required for the joint task is 0.71022 s, and the joint angle trajectory planning time is 0.13634 s. From Fig. 4, it can be seen that the whole trajectory planning of the mission space is smooth, the angle space trajectory planning is direct, the trajectory changes greatly, and has certain impact on the manipulator.

#### Time optimization of vibration trajectory based on I-PSO algorithm

##### Particle swarm optimization

Particle Swarm Optimization (PSO) is a swarm-intelligent global stochastic search algorithm that simulates flocks' migration, foraging behavior, and fuses concepts of social psychology such as individual cognition and social influence^18^, a member of Evolutionary Algorithm-EA, abbreviated PSO^19^.

Particle swarm velocity update Formula:

1 $${V}_{id}^{k+1}=W{V}_{id}^{k}+{C}_{1}{r}_{1}({P}_{id.{\text{pbest}}}^{k}-{X}_{id}^{k})+{C}_{2}{r}_{2}({P}_{d.{\text{gbest}}}^{k}-{X}_{id}^{k})$$

$i$: Particle number; $d$: Particle dimension number; $k$: Number of iterations; $W$:Inertia weight; ${c}_{1}$: Individual learning factors; ${c}_{2}$: Group learning factor; ${r}_{1},{r}_{2}$: A random number in an interval [0, 1]; ${V}_{id}^{k}$: The $d$-dimensional velocity vector of particle $i$ in the $k$-th iteration; ${X}_{id}^{k}$: The $d$-dimensional position vector of particle $i$ in the $k$-th iteration; ${P}_{id.{\text{pbest}}}^{k}$: The historical optimal position of particle $i$ in $d$-dimension in the $k$-th iteration; ${p}_{d.{\text{gbest}}}^{k}$: The historical optimal position of the $d$-dimension in the $k$-th iteration.

$W{V}_{id}^{k}$ (Inertial part): by the inertia weight and the particle's speed, indicating the particle's previous state of trust in their motion. ${C}_{1}{r}_{1}({P}_{id.{\text{pbest}}}^{k}-{X}_{id}^{k})$ (Individual Cognition): the self-cognition of a particle, that is, the part of the particle's own experience, which can be understood as the distance and direction between the current position of the particle and the optimal position in the individual's history. ${C}_{2}{r}_{2}({P}_{d.\text{g best}}^{k}-{X}_{id}^{k})$ (Social sharing): information sharing and cooperation between particle swarm, that is, the experience of optimization from other particles in the swarm, can be understood as the distance and direction between the current position of the particle and the historical optimal position of the swarm.

##### Improved particle swarm optimization algorithm (I-PSO)

The inertia weight w and learning factors ${C}_{1}$, ${C}_{2}$, and other parameters of the traditional particle swarm optimization algorithm are fixed. In the optimization process, the algorithm is prone to fall into the local optimal trap and other problems, resulting in slow convergence, local optimization imbalance, and global optimization errors^20^. When using the PSO algorithm to plan the optimal time of the manipulator, it is necessary to ensure the constraint of the velocity boundary of the manipulator's joint and the continuity of the acceleration. Therefore, in this paper, the motion time of the manipulator is optimized by introducing adaptive inertia weight, asynchronous learning factor, and 3–5–3 polynomial interpolation Particle Swarm Optimization. The improved particle swarm optimization algorithm is abbreviated as I-PSO.

Adaptive inertia weight $W$:

2 $$W={W}_{max}-({W}_{max}-{W}_{min}){e}^{({\frac{4k}{{N}_{max}})}^{2}}$$

$k, {N}_{max}$: The current and maximum values for the number of iterations, respectively; ${W}_{max}, {W}_{min}$: The maximum and the minimum of the inertia weight.

In particle swarm optimization, inertia weight W can balance the global and local optimization ability and convergence speed by controlling the size of search area^21^. When a larger inertia weight W is used, the inertia of particle motion and the ability of searching the extended space are enhanced, which is beneficial to the global optimization, jumping out of the local extremum and not falling into the local optimum, the local optimization ability will be enhanced, which enables the algorithm to converge to the optimal solution quickly^22^. The traditional PSO algorithm only increases or decreases the inertia weight, and cannot cope with the changing demand in the complex real-life environment. Therefore, this paper introduces an adaptive inertia weight that can achieve different weights at different stages according to the complex search case, as shown in Formula (2). In the early stage of the PI-PSO algorithm, due to the large problem space, to ensure the balance of search speed and precision, the larger inertia weight is used in the early stage of PI-PSO algorithm to reach the higher global search ability to obtain the correct solution. At a later stage, the local search capability is improved by using smaller inertia weights to improve convergence accuracy.

Asynchronous learning factor ${C}_{1}$, ${C}_{2}$:

3 $${C}_{1}=\frac{({C}_{1f}-{C}_{1i})k}{{N}_{max}}+{C}_{1i}$$

4 $${C}_{2}=\frac{({C}_{2f}-{C}_{2i})k}{{N}_{max}}+{C}_{2i}$$

${C}_{1}$: Acceleration coefficient of individual cognition; ${C}_{2}$: Accelerated coefficient of social sharing.

From the Formula (1), we know that the learning factor ${C}_{1}$ affects the “Individual cognition” ability of the particle, and it should decrease gradually with the increase of the number of search iterations, while the learning factor ${C}_{2}$ affects the “Social sharing” ability of the particle, it should increase as the number of search iterations increases. Since the learning factors ${C}_{1}$ and ${C}_{2}$ of traditional particle swarm optimization are fixed, this will affect the algorithm's optimization speed and solution accuracy balance. Therefore, asynchronous learning factors are introduced in this paper, as shown in Formulas (3) and (4). In the early stage of optimization, the improved algorithm ensures that the region of the initial individual cognitive solution is larger than that of the group cognitive solution and that the particles do not fall into the local extremum trap, it makes the group have a strong global optimization ability and convergence speed.

3–5–3 Polynomial interpolation:

5 $${h}_{i1}(t){h}_{i1}(t)={a}_{i13}{t}^{3}+{a}_{i12}{t}^{2}+{a}_{i11}{t}^{1}+{a}_{i10}$$

6 $${h}_{i2}(t)={a}_{i25}{t}^{5}+{a}_{i24}{t}^{4}+{a}_{i23}{t}^{3}+{a}_{i22}{t}^{2}+{a}_{i21}{t}^{1}+{a}_{i20}$$

7 $${h}_{i3}(t)={a}_{i33}{t}^{3}+{a}_{i32}{t}^{2}+{a}_{i31}{t}^{1}+{a}_{i30}$$

${h}_{im}(t)$: The trajectory function of the $m$ time segment of the $i$ joint; ${a}_{imj}$ represents the $j$-segment coefficient of the $m$-segment interpolation function for the $i$-segment articulation trajectory. The matrix form of the coefficient is shown in Formula (9), and Formula (8) is the coefficient solution.

8 $$b={[0, 0, 0, 0, 0, 0, {X}_{3}, 0 , 0 ,{ X}_{0} , 0 , 0,{ X}_{2},{ X}_{1}]}^{T}$$

9 $$A=\left[\begin{array}{c}{t}_{1}^{3}\\ {3t}_{1}^{2}\\ {6t}_{1}\\ 0\\ 0\\ 0\\ 0\\ 0\\ 0\\ 0\\ 0\\ 0\\ 0\\ 0\end{array} \begin{array}{c}{t}_{1}^{2}\\ 2{t}_{1}\\ 2\\ 0\\ 0\\ 0\\ 0\\ 0\\ 0\\ 0\\ 0\\ 1\\ 0\\ 0\end{array} \begin{array}{c}{t}_{1}\\ 1\\ 0\\ 0\\ 0\\ 0\\ 0\\ 0\\ 0\\ 0\\ 1\\ 0\\ 0\\ 0\end{array} \begin{array}{c}1\\ 0\\ 0\\ 0\\ 0\\ 0\\ 0\\ 0\\ 0\\ 1\\ 0\\ 0\\ 0\\ 0\end{array} \begin{array}{c}0\\ 0\\ 0\\ {t}_{2}^{5}\\ {5t}_{2}^{4}\\ {20t}_{2}^{3}\\ 0\\ 0\\ 0\\ 0\\ 0\\ 0\\ 0\\ 0\end{array} \begin{array}{c}0\\ 0\\ 0\\ {t}_{2}^{4}\\ {4t}_{2}^{3}\\ {12t}_{2}^{2}\\ 0\\ 0\\ 0\\ 0\\ 0\\ 0\\ 0\\ 0\end{array} \begin{array}{c}0\\ 0\\ 0\\ {t}_{2}^{3}\\ {3t}_{2}^{2}\\ {6t}_{2}\\ 0\\ 0\\ 0\\ 0\\ 0\\ 0\\ 0\\ 0\end{array} \begin{array}{c}0\\ 0\\ -2\\ {t}_{2}^{2}\\ {2t}_{2}\\ 2\\ 0\\ 0\\ 0\\ 0\\ 0\\ 0\\ 0\\ 0\end{array} \begin{array}{c}0\\ -1\\ 0\\ {t}_{2}\\ 1\\ 0\\ 0\\ 0\\ 0\\ 0\\ 0\\ 0\\ 0\\ 0\end{array} \begin{array}{c}-1\\ 0\\ 0\\ 1\\ 0\\ 0\\ 0\\ 0\\ 0\\ 0\\ 0\\ 0\\ 0\\ 1\end{array} \begin{array}{c}0\\ 0\\ 0\\ 0\\ 0\\ 0\\ {t}_{3}^{3}\\ {3t}_{3}^{2}\\ {6t}_{3}\\ 0\\ 0\\ 0\\ 0\\ 0\end{array} \begin{array}{c}0\\ 0\\ 0\\ 0\\ 0\\ -2\\ {t}_{3}^{2}\\ {2t}_{3}\\ 2\\ 0\\ 0\\ 0\\ 0\\ 0\end{array} \begin{array}{c}0\\ 0\\ 0\\ 0\\ -1\\ 0\\ {t}_{3}\\ 1\\ 0\\ 0\\ 0\\ 0\\ 0\\ 0\end{array} \begin{array}{c}0\\ 0\\ 0\\ -1\\ 0\\ 0\\ 1\\ 0\\ 0\\ 0\\ 0\\ 0\\ 1\\ 0\end{array}\right]$$

10 $$B= inv(A)$$

11 $$H=B*b$$

b: A coefficient containing an unknown number; A: A simple function of time; H: The motion matrix of the manipulator with respect to time T.

##### (I-PSO) algorithm target content description (flowchart)

**Figure 5:**
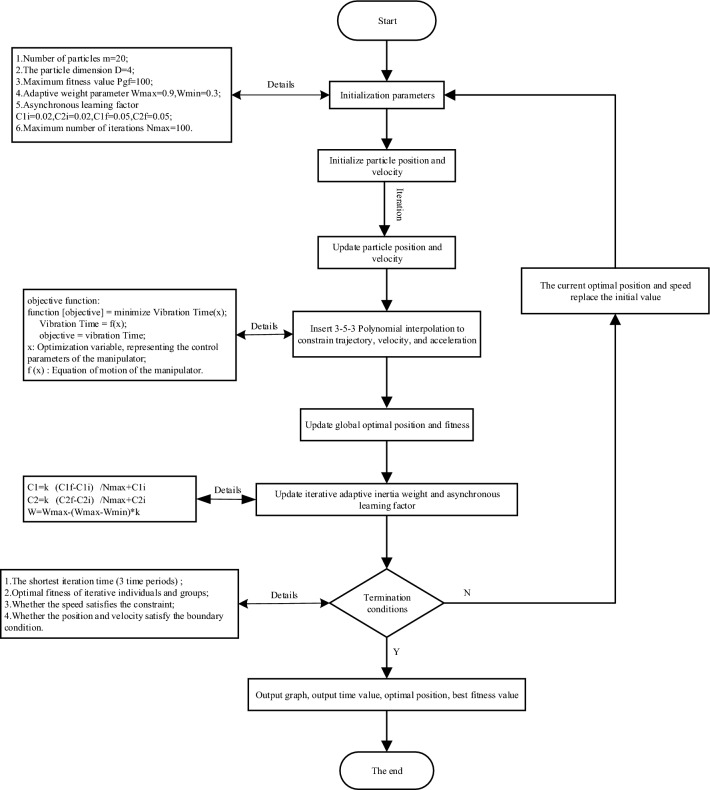
I-PSO algorithm flow chart.

The flow chart of the improved I-PSO algorithm is shown in Fig. 5. The I-PSO algorithm is based on traditional particle swarm optimization (PSO-RRB-algorithm). By improving adaptive inertia weight and asynchronous learning factor, Polynomial interpolation between optimization speed and precision can be ensured.

### Methodology statement

This study states that the experiment and simulation of the vibration trajectory of the manipulator, including the collection of Hippophae rhamnoides materials, are in line with relevant institutions, national and international standards.

## Results

**Figure 6:**
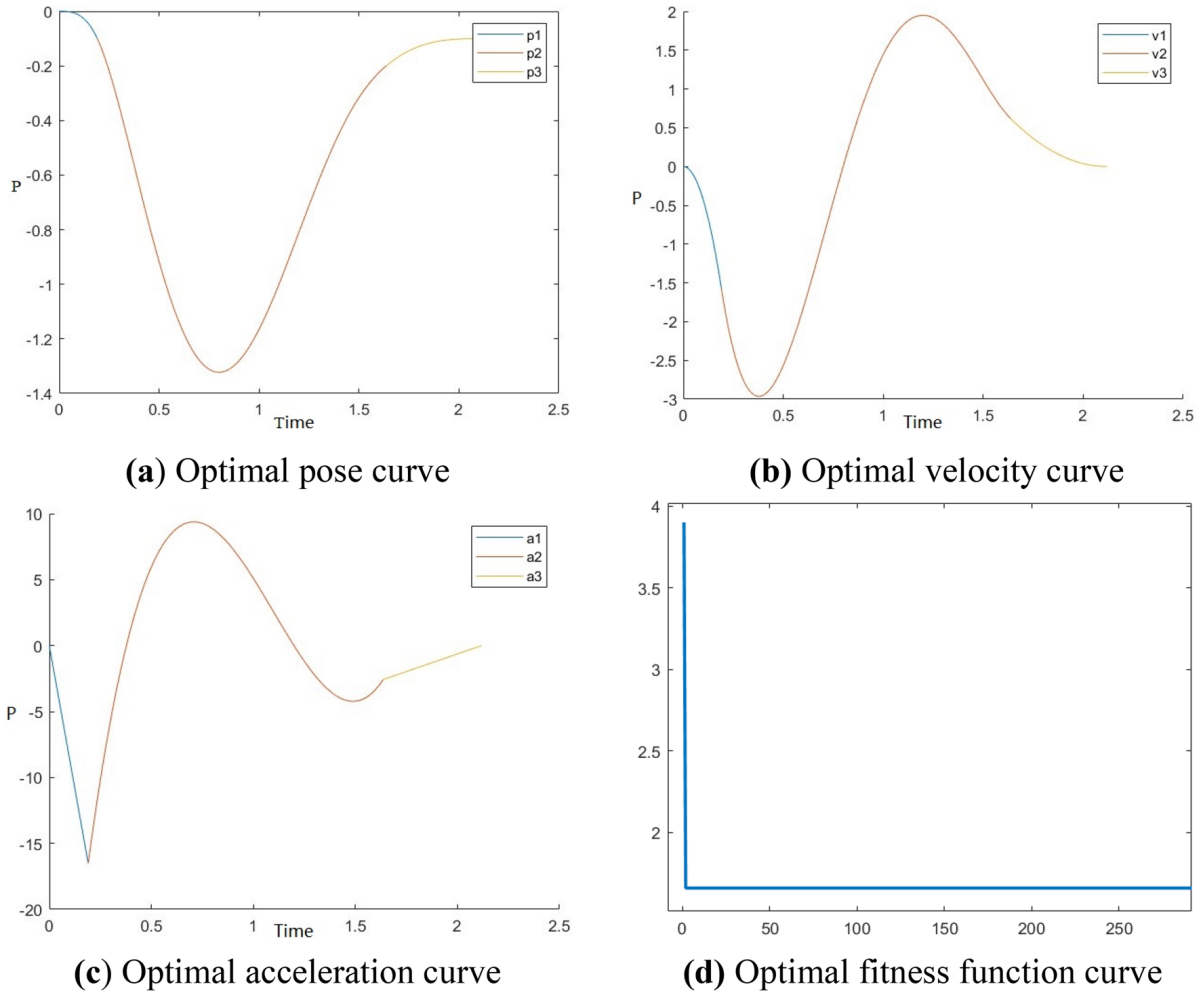
Optimal time trajectory planning of manipulator on X-axis.

**Figure 7:**
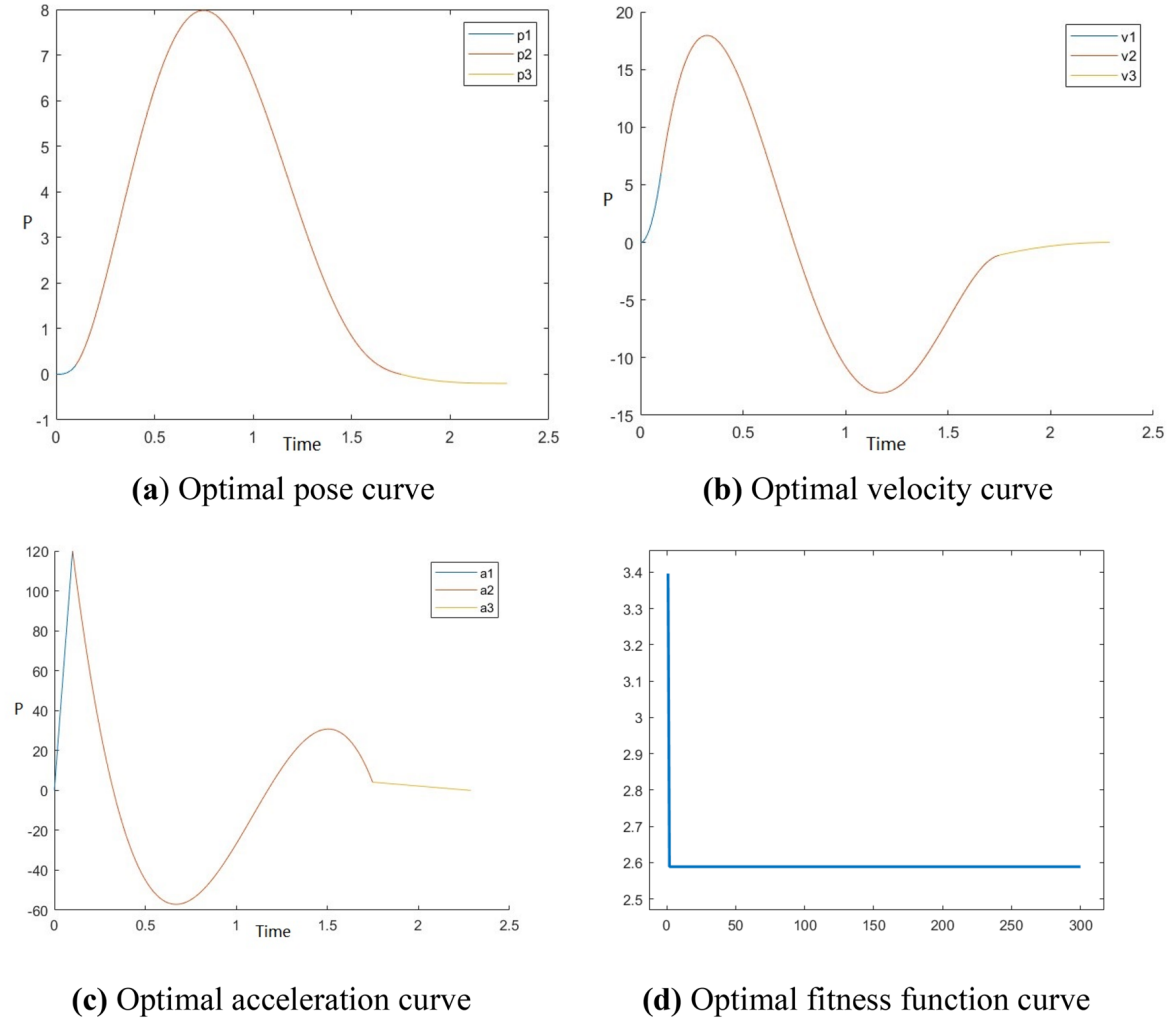
Optimal time trajectory planning of manipulator on Y-axis.

**Figure 8:**
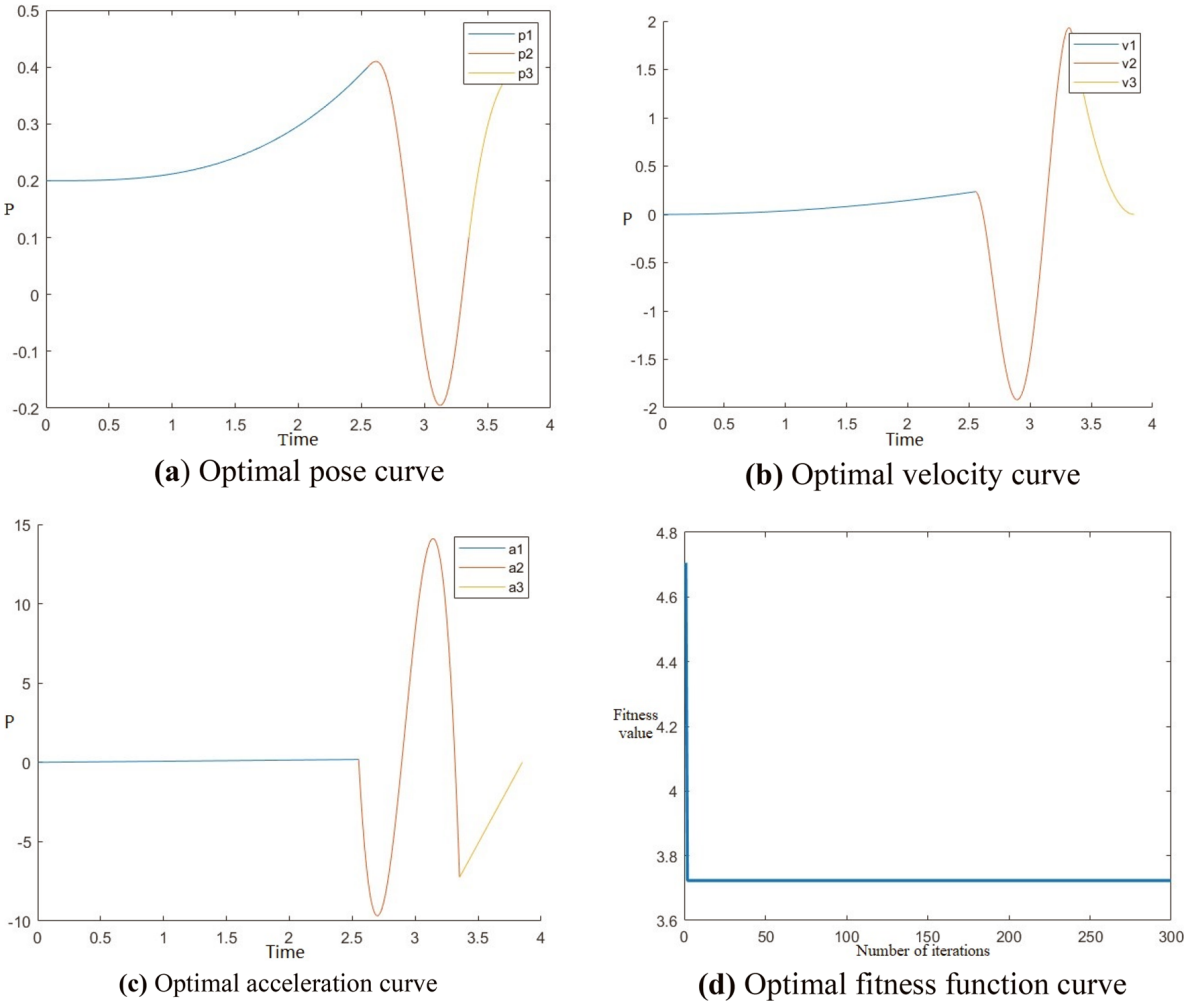
Optimal time trajectory planning of manipulator on Z-axis.

According to the experimental data of Supplementary materials, the optimization results of each axis near the optimal time in the simulation experiment are shown as follows: the trajectory planning of the manipulator on the X-axis by multi-group simulation experiments is closest to the experimental average value as shown in Fig. 6. The trajectory planning of the manipulator on the Y-axis is most close to the experimental average through several simulation experiments, as shown in Fig. 7. The trajectory planning of the manipulator on the Z-axis is most close to the experimental average through several simulation experiments, as shown in Fig. 8.

When the time of the vibration trajectory of the manipulator is optimized by the algorithm, the pose of the X-axis and the Z-axis fluctuates greatly. Four groups of simulation experiments are carried out for optimal time trajectory planning. The results show that one group of experimental data is unqualified, and the remaining six groups of optimal time are 0.396 s, 0.483 s, 0.735 s and 0.700 s, 0.408 s, 0.498 s. Three groups of simulation experiments were carried out on the Y-axis trajectory, and the optimal time was 0.802 s, 0.539 s, and 0.533 s. The average optimal time of the X-axis is 0.538 s; the average optimal time of the Y-axis is 0.536 s; the average optimal time of the Z-axis is 0.535 s.

To verify the validity and reliability of the algorithm, the vibration trajectories of Seabuckthorn branches were analyzed by the method of variance analysis. The fruit data of Hippophae rhamnoides L. were obtained as shown in Table 1.

**Table 1:** Experimental data of Hippophae rhamnoides L.

No.	Vibration frequency (Hz)	Amplitude of vibration (mm)	Vibration time (s)	Fruit drop rate	Average fruit drop rate	Optimize the number of fruit drop before	Average fruit drop rate before optimization
1	21.1	30	2	98.84%	98.21%	96.51%	97.05%
2	21.1	30	2	97.59%		97.59%	
3	21.1	30	4	98.94%	99.47%	97.87%	98.31%
4	21.1	30	4	100.00%		98.75%	
5	21.1	20	4	96.51%	96.76%	96.51%	97.26%
6	21.1	20	4	97.00%		98.00%	
7	16.2	30	4	96.55%	96.08%	95.40%	94.40%
8	16.2	30	4	95.60%		93.41%	
9	16.2	30	4	96.70%	96.29%	95.60%	95.74%
10	16.2	30	4	95.88%		95.88%	
11	16.2	20	2	95.51%	95.43%	94.38%	94.87%
12	16.2	20	2	95.35%		95.35%	
13	14.4	30	4	94.62%	95.18%	93.55%	94.65%
14	14.4	30	4	95.74%		95.74%	
15	14.4	30	2	94.74%	94.14%	94.74%	92.53%
16	14.4	30	2	93.55%		90.32%	
17	14.4	20	4	93.81%	94.16%	92.78%	93.10%
18	14.4	20	4	94.51%		93.41%	

SPSS software was used to analyze the fruit drop rate variance, and test tables of inter-body effects were shown in Table 2.

**Table 2:** Inter-body effect test of fruit drop rate.

Source	Class III sum of squares	Degree of freedom	Mean square	F	P
Modify the model	0.003^a^	7	0	159.763	0.061
Intercept	7.029	1	7.029	3,135,621.505	0
Vibration frequency (Hz)	0.002	2	0.001	413.748	0.035
Amplitude of vibration/mm	0	1	0	155.711	0.051
Vibration time (s)	0	1	0	58.787	0.083
Error	2.24E–06	1	2.24E–06		
Total	8.33	9			
Corrected total	0.003	8			

^a^R Square = 0.999(Adjusted R Square = 0.993).

In Table 2, the significance of the modified model is greater than 0.05, so there is no significant difference between the modified model and the whole analysis of the variance model, and the effect is vibration frequency > vibration amplitude > vibration time.

## Conclusion

Based on the 3–5–3 Polynomial interpolation function, the vibration trajectory of Hippophae rhamnoides is optimized in time by using an improved Particle swarm optimization system (I-PSO), the results of the simulation experiment are as follows:

1. In this study, the trajectory planning of mission space can realize the drop of branches and leaves in the process of picking up and can ensure the continuity of joint pose, velocity, and acceleration of the trajectory, much closer to the actual movement of the robot.
2. In this study, an improved particle swarm optimization (PSO-RRB-algorithm combined with 3–5–3 Polynomial interpolation is used to select the optimal time for the manipulator to complete the motion in the shortest time and satisfy the speed constraint, the simulation results show that the average optimal linkage time is 0.536539094 s, which is 24.5% higher than the original trajectory time 0.71022 s planned by JTRAJ function. The effectiveness and superiority of the I-PSO algorithm for time trajectory optimization are proved. Contribute to the development of research on trajectory planning and optimization of related manipulators. The utility model can be used for reducing the movement time of the mechanical arm and improving the work efficiency of seabuckthorn fruit collection. The optimization of traditional algorithms is convenient to promote the development of artificial intelligence. The trajectory also ensures the continuity of the robot's terminal posture.
3. SPSS was used to analyze the variance of the vibration orthogonal test of seabuckthorn branches. The data show that the average fruit drop rate of the optimized algorithm can reach 96.19%, which is 0.87% higher than the 95.32% average fruit drop rate of the original JTRAJ function trajectory planning, the validity and reliability of I-PSO algorithm for optimal time planning of seabuckthorn fruit separation vibration trajectory were verified. But from the experimental data, we can see that the acceleration changes more quickly will have an impact, the later need to study from this aspect.

### Supplementary Information

Supplementary Figures.

## Data Availability

All data generated or analyzed during this study are included in this published article and its Supplementary information files.
