# Supplementary material for: Trajectory analysis and optimization of sea buckthorn fruit vibration separation manipulator based on I-PSO algorithm
Source: Sci Rep. 2023 Nov 17;13:20124. doi: 10.1038/s41598-023-47001-2 (PMC10656555; doi:10.1038/s41598-023-47001-2)
Supplement: Supplementary file 1 — Supplementary Figures. [file 41598_2023_47001_MOESM1_ESM.zip › Supplementary Information.docx]

**1. Kinematics state analysis of manipulator**

When analyzing the trajectory of a manipulator, it is necessary to transform the coordinates of each joint into a homogeneous transformation matrix, as shown in Formula (1). The transformation of the matrix represents the translation and rotation of the trajectory. The position and orientation of the end of the manipulator can be obtained by forward-solving the homogeneous matrix.

The research object of this paper is a six-of serial manipulator, as shown in Figure 1. From the Kinematics, you can draw the coordinates of each joint of the robot arm, as shown in Figure 2.


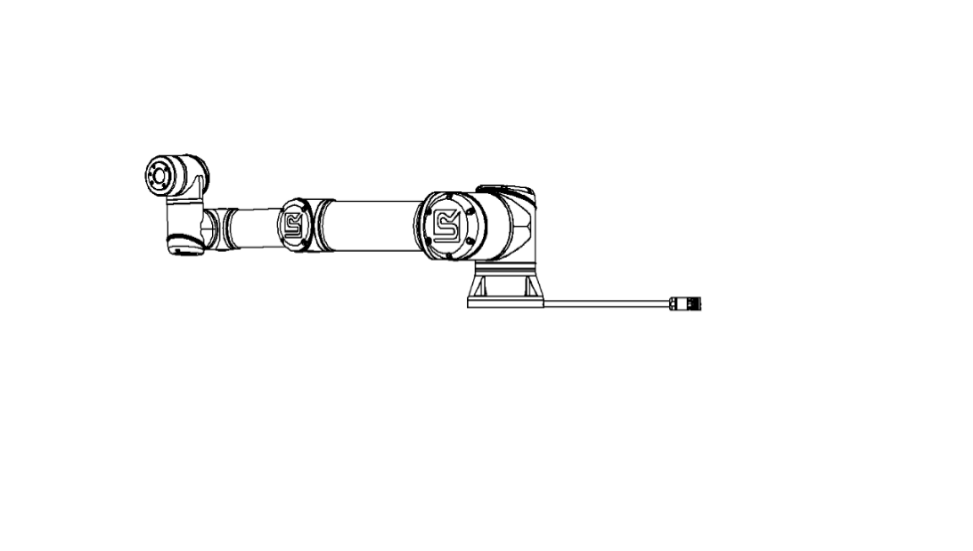

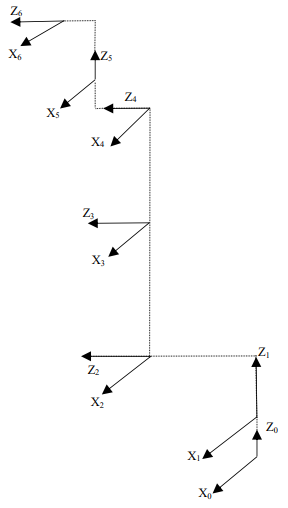


**Figure 1.** Schematic diagram of the robot arm

**Figure 2.** Coordinates of each joint of the manipulator

In this paper, it is necessary to determine the angular value of the vibration trajectory. The detailed kinematics analysis and solution process are as follows:

${}_{i}^{i-1}T=T_{\hat{X}_{i-1}}(\alpha_{i-1})T_{\hat{X}_{R}}(a_{i-1})T_{\hat{Z}_{Q}}(\theta_{i})T_{\hat{Z}_{p}}(d_{i})=\left[ \begin{matrix} c\theta_{i} & -s\theta_{i} & 0 & a_{i-1} \\ s\theta_{i}c\alpha_{i-1} & c\theta_{i}c\alpha_{i-1} & -s\alpha_{i-1} & -s\alpha_{i-1}d_{i} \\ s\theta_{i}s\alpha_{i-1} & c\theta_{i}s\alpha_{i-1} & c\alpha_{i-1} & c\alpha_{i-1}d_{i} \\ 0 & 0 & 0 & 1 \end{matrix} \right]$ (1)

### $a_{i}$: The vertical length between two-rod shafts; $\alpha_{i}$: Torsion angle of two adjacent connecting rods; $d_{i}$: The offset between the vertical axis and the connecting rod; $\theta_{i}$: The angle of the perpendicular line of the two joint axes.

${}_{6}^{0}T={}_{1}^{0}T\times_{2}^{1}T\times_{3}^{2}T\times_{4}^{3}T\times_{5}^{4}T\times_{6}^{5}T=T_{tool}=\left[ \begin{matrix} n_{x} & o_{x} & a_{x} & p_{x} \\ n_{y} & o_{y} & a_{y} & p_{y} \\ n_{z} & o_{z} & a_{z} & p_{z} \\ 0 & 0 & 0 & 1 \end{matrix} \right]$ (2)

${}_{6}^{0}T$: It represents the homogeneous transformation matrix of the end position of the manipulator after being moved and rotated in the absolute coordinate system;

$\begin{matrix} n_{x} & n_{y} & n_{z} \end{matrix}$: The dynamic coordinates of the manipulator are relative to the X-axis rotated directional offset coordinates in the absolute coordinate system;

$\begin{matrix} o_{x} & o_{y} & o_{z} \end{matrix}$: The dynamic coordinates of the manipulator are relative to the directional offset coordinates rotated along the Y-axis in the absolute coordinate system;

$\begin{matrix} a_{x} & a_{y} & a_{z} \end{matrix}$: The dynamic coordinates of the manipulator are relative to the directional offset coordinates rotated along the Z-axis in the absolute coordinate system;

$\begin{matrix} p_{x} & p_{y} & p_{z} \end{matrix}$: The dynamic coordinates of the manipulator are moved relative to the coordinates in the absolute coordinate system;

The transformation matrix T of the end position of the manipulator was obtained by forward kinematics, and the joint angles were obtained by inverse kinematics.

${}_{6}^{1}T=({{}_{1}^{0}{T)}}^{-1}\times_{6}^{0}T=({{}_{1}^{0}{T)}}^{-1}\times\left[ \begin{matrix} n_{x} & o_{x} & a_{x} & p_{x} \\ n_{y} & o_{y} & a_{y} & p_{y} \\ n_{z} & o_{z} & a_{z} & p_{z} \\ 0 & 0 & 0 & 1 \end{matrix} \right]$ (3)

Both sides of the equation are multiplied together $({{}_{1}^{0}{T)}}^{-1}$:

${}_{6}^{1}T=\left[ \begin{matrix} C_{234}C_{5}C_{6}-S_{234}S_{6} & -C_{234}C_{5}C_{6}-S_{234}C_{6} & C_{234}S_{5} & a_{3}C_{23}+a_{2}C_{2}+d_{5}S_{234}-d_{6}C_{234}S_{5} \\ -S_{5}S_{6} & S_{5}S_{6} & -C_{5} & -d_{6}C_{5}-d_{4} \\ S_{234}C_{5}C_{6}+C_{234}S_{6} & C_{234}C_{6}-S_{234}C_{5}S_{6} & S_{234}S_{5} & a_{3}S_{23}+d_{2}C_{2}-d_{5}C_{234}-d_{6}S_{234}S_{5} \\ 0 & 0 & 0 & 1 \end{matrix} \right]$

$=\left[ \begin{matrix} C_{1}n_{x}+S_{1}n_{y} & C_{1}o_{x}+S_{1}o_{y} & C_{1}a_{x}+S_{1}a_{y} & C_{1}p_{x}+S_{1}p_{y} \\ -S_{1}n_{x}+C_{1}n_{y} & -S_{1}o_{x}+C_{1}o_{y} & -S_{1}a_{x}+C_{1}a_{y} & -S_{1}p_{x}+C_{1}p_{y} \\ n_{z} & o_{z} & a_{z} & p_{z}-d_{1} \\ 0 & 0 & 0 & 1 \end{matrix} \right]$ (4)

$\theta=\mathrm{atan}2\left( p_{y},p_{x} \right)-\mathrm{atan}2\left( -d,\pm\sqrt{{p_{x}}^{2}+{p_{y}}^{2}-d^{2}} \right)$ ${p_{x}}^{2}+{p_{y}}^{2}-d^{2}\geq0$ (5)

Move first, the first three angles are moving angles, let $\theta_{1},\theta_{2},\theta_{3}$ layer decomposition:

$${}_{i}^{i-1}T=T_{\hat{X}_{i-1}}\left( \alpha_{i-1} \right)T_{\hat{X}_{R}}\left( a_{i-1} \right)T_{\hat{Z}_{Q}}\left( \theta_{i} \right)T_{\hat{Z}_{p}}\left( d_{i} \right)$$

$=\left[ \begin{matrix} c\theta_{i} & -s\theta_{i} & 0 & a_{i-1} \\ s\theta_{i}c\alpha_{i-1} & c\theta_{i}c\alpha_{i-1} & -s\alpha_{i-1} & -s\alpha_{i-1}d_{i} \\ s\theta_{i}s\alpha_{i-1} & c\theta_{i}s\alpha_{i-1} & c\alpha_{i-1} & c\alpha_{i-1}d_{i} \\ 0 & 0 & 0 & 1 \end{matrix} \right]\left[ \begin{aligned} a_{i-1} \\ -s\alpha_{i-1}d_{i} \\ c\alpha_{i-1}d_{i} \\ 1 \end{aligned} \right]$

$=\left[ \begin{aligned} x \\ y \\ z \\ 1 \end{aligned} \right]=^{0}P_{4org}=_{1}^{0}T_{2}^{1}T_{3}^{2}T^{3}P_{4org}={{}_{1}^{0}T}_{2}^{1}T_{3}^{2}T\left[ \begin{aligned} a_{3} \\ -d_{4}s\alpha_{3} \\ d_{4}c\alpha_{3} \\ 1 \end{aligned} \right]$

$={{}_{1}^{0}T}_{2}^{1}T\left[ \begin{aligned} f_{1}(\theta_{3}) \\ f_{2}(\theta_{3}) \\ f_{3}(\theta_{3}) \\ 1 \end{aligned} \right]\left[ \begin{aligned} f_{1}(\theta_{3}) \\ f_{2}(\theta_{3}) \\ f_{3}(\theta_{3}) \\ 1 \end{aligned} \right]={}_{3}^{2}T\left[ \begin{aligned} a_{3} \\ -d_{4}s\alpha_{3} \\ d_{4}c\alpha_{3} \\ 1 \end{aligned} \right]$ (6)

Decomposition: $f_{i}(\theta_{3})$ is a function of $\theta_{3}$:

$f_{1}(\theta_{3})=a_{3}c_{3}+d_{4}s\alpha_{3}s_{3}+a_{2}$ (7)

$f_{2}(\theta_{3})=a_{3}c\alpha_{2}s_{3}-d_{4}s\alpha_{3}c\alpha_{2}c_{3}-d_{4}s\alpha_{2}c\alpha_{3}-d_{3}s\alpha_{2}$ (8)

$f_{3}(\theta_{3})=a_{3}s\alpha_{2}s_{3}-d_{4}s\alpha_{3}s\alpha_{2}c_{3}+d_{4}c\alpha_{2}c\alpha_{3}+d_{3}c\alpha_{2}$ (9)

${0_{P}}_{4org}=\left[ \begin{aligned} x \\ y \\ z \\ 1 \end{aligned} \right]={{}_{1}^{0}T}_{2}^{1}T\left[ \begin{aligned} f_{1}(\theta_{3}) \\ f_{2}(\theta_{3}) \\ f_{3}(\theta_{3}) \\ 1 \end{aligned} \right]={}_{1}^{0}T\left[ \begin{aligned} g_{1}(\theta_{2},\theta_{3}) \\ g_{2}(\theta_{2},\theta_{3}) \\ g_{3}(\theta_{2},\theta_{3}) \\ 1 \end{aligned} \right]=\left[ \begin{aligned} c_{1}g_{1}-s_{1}g_{2} \\ s_{1}g_{1}+c_{1}g_{2} \\ g_{3} \\ 1 \end{aligned} \right]$ (10)

$g_{i}(\theta_{2},\theta_{3})$ is a function of $\theta_{2},\theta_{3}$:

$g_{1}(\theta_{2},\theta_{3})=c_{2}f_{1}-s_{2}f_{2}+a_{1}$ (11)

$g_{2}(\theta_{2},\theta_{3})=s_{2}c\alpha_{1}f_{1}+c_{2}c\alpha_{1}f_{2}-s\alpha_{1}f_{3}-d_{2}s\alpha_{1}$ (12)

$g_{3}(\theta_{2},\theta_{3})=s_{2}s\alpha_{1}f_{1}+c_{2}s\alpha_{1}f_{2}+c\alpha_{1}f_{3}+d_{2}c\alpha_{1}$ (13)

$r$ is a function of $\theta_{2},\theta_{3}$:

$r=f_{1}^{2}+f_{2}^{2}+f_{3}^{2}+a_{1}^{2}+d_{2}^{2}+2d_{2}f_{3}+2a_{1}(c_{2}f_{1}-s_{2}f_{2})=(k_{1}c_{2}+k_{2}s_{2})2a_{1}+k_{3}$ (14)

$k_{1}(\theta_{3})=f_{1}$ (15)

$k_{2}(\theta_{3})=-f_{2}$ (16)

$k_{3}(\theta_{3})=f_{1}^{2}+f_{2}^{2}+f_{3}^{2}+a_{1}^{2}+d_{2}^{2}+2d_{2}f_{3}$ (17)

$z$ is a function of $\theta_{2},\theta_{3}$:

$z=g_{3}=(k_{1}s_{2}-k_{2}c_{2})s\alpha_{1}+k_{4}$ (18)

$k_{4}(\theta_{3})=f_{3}c\alpha_{1}+d_{2}c\alpha_{1}$ (19)

$\{\begin{aligned} r=(k_{1}c_{2}+k_{2}s_{2})2a_{1}+k_{3} \\ z=(k_{1}s_{2}-k_{2}c_{2})s\alpha_{1}+k_{4} \end{aligned}$ (20)

If $a_{1}=0$

$r=k_{3}(\theta_{3})=f_{1}^{2}+f_{2}^{2}+f_{3}^{2}+a_{1}^{2}+d_{2}^{2}+2d_{2}f_{3}$ (21)

If $s\alpha_{1}=0$

$z=k_{4}(\theta_{3})=f_{3}c\alpha_{1}+d_{2}c\alpha_{1}$ (22)

Else:

$\frac{(r-k_{3})^{2}}{4\alpha_{1}^{2}}+\frac{(z-k_{4})^{2}}{s^{2}\alpha_{1}}=k_{1}^{2}+k_{2}^{2}$ (23)

By finding $\theta_{3}$ in all three cases, $f_{i}$ and $k_{i}$ are known. Put $f_{i}$ and $k_{i}$ into the following formula to find $\theta_{2}$.

$r=g_{1}^{2}+g_{2}^{2}+g_{3}^{2}=(k_{1}c_{2}+k_{2}s_{2})2a_{1}+k_{3}$ (24)

Immediately after that, you know the value of $g_{i}$, and you get the value of $\theta_{1}$.

$x=c_{1}g_{1}(\theta_{2},\theta_{3})-s_{1}g_{2}(\theta_{2},\theta_{3})$ (25)

The rotation matrix of Z-Y-Z Euler Angles is used to solve $\theta_{4},\theta_{5},\theta_{6}$.

${}_{6}^{3}R={}_{3}^{0}R{}_{3}^{-10}R$ (26)

${{}_{B}^{A}R}_{Z^{'}Y^{'}Z^{'}}\left( \gamma,\beta,\alpha\right)=\left[ \begin{matrix} cac\beta c\gamma-sas\gamma& -cac\beta s\gamma-sac\gamma& cas\beta\\ sac\beta c\gamma+cas\gamma& -sac\beta s\gamma+cac\gamma& sas\beta\\ -s\beta c\gamma& s\beta s\gamma& c\beta\end{matrix} \right]$ (27)

### **2.Parameter setting**

### The research object of this paper is a six-dof serial manipulator, and the DH parameter table is established, as shown in Table 1. The joint coordinates are transformed into a homogeneous matrix, as shown in Formula (1).

### **Table 1.** D-H parameter table

| $i$ | $\alpha_{i-1}$ | $a_{i-1}$ | $d_{i}$ | $\theta_{i}$ |
| --- | --- | --- | --- | --- |
| 1 | 0 | 0.00 | 180.50 | $\theta_{1}$ (0) |
| 2 | 90 | 0.00 | 270.89 | $\theta_{2}$ (90) |
| 3 | 0 | 478.40 | 0.00 | $\theta_{3}$ (0) |
| 4 | 0 | 360.05 | 0.00 | $\theta_{4}$ (0) |
| 5 | -90 | 0.00 | 174.32 | $\theta_{5}$ (-90) |
| 6 | 90 | 0.00 | 173.57 | $\theta_{6}$ (0) |

According to the DH parameters of the manipulator, a three-dimensional kinematics model of the manipulator is established. The range of the joint rotation is shown in Table 2 by simulation in SolidWorks. The operating range of the manipulator can be determined by the QLIM function. As shown in Figure 3. The trajectories planned simultaneously with the JTRAJ function are shown in Figure 4.

### **Table 2.** Range of joint rotation

| Joins $i$ | [$i_{min},i_{max}$]/($^{\circ}$) |
| --- | --- |
| 1,4,5,6 | [-180,180] |
| 2 | [0,180] |
| 3 | [-70,100] |

###
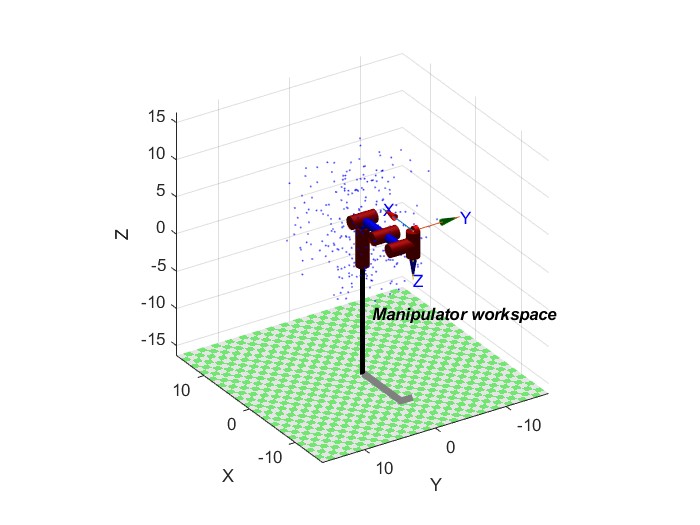

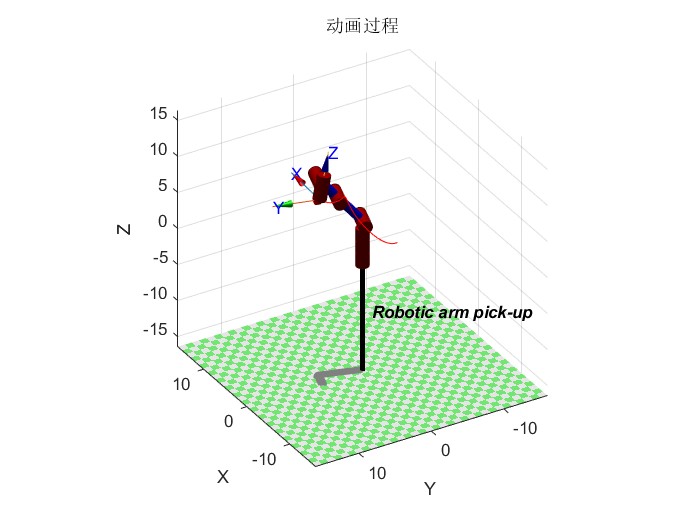


### **Figure 3.** The 3D model and operating range of the manipulator are established by using the algorithm.

**Figure 4.** JTRAJ function planning manipulator to pick up Seabuckthorn branch path.

**3. Simulation results**

Specific experiments can be seen in figure 5 ~ 16.


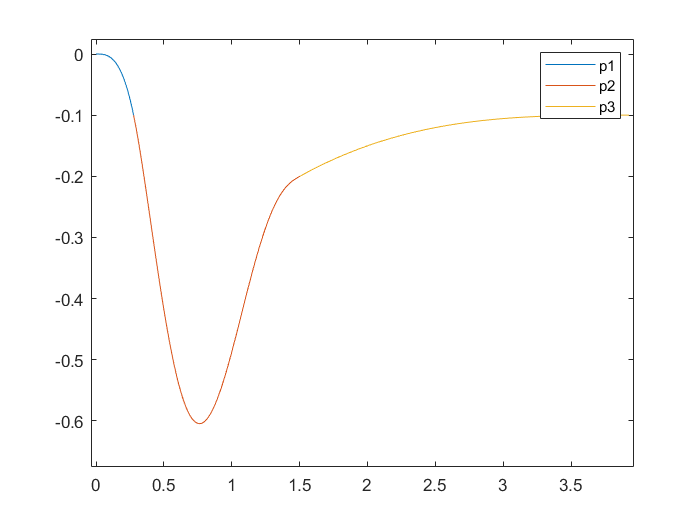

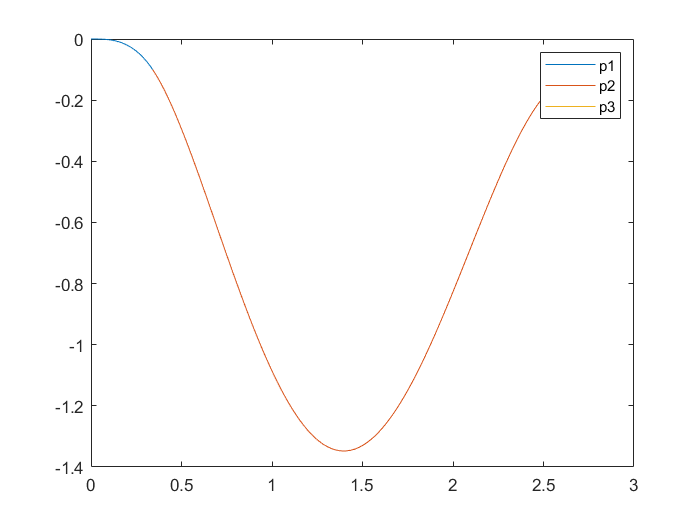


**(a)** Simulation Experiment 1

**(b)** Simulation Experiment 2


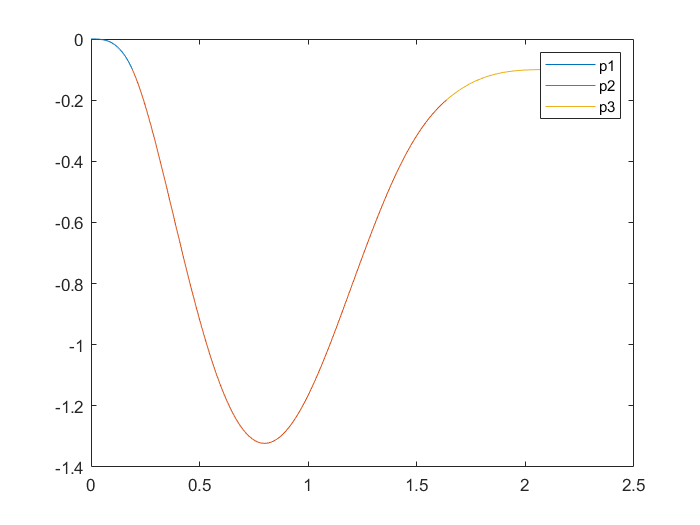

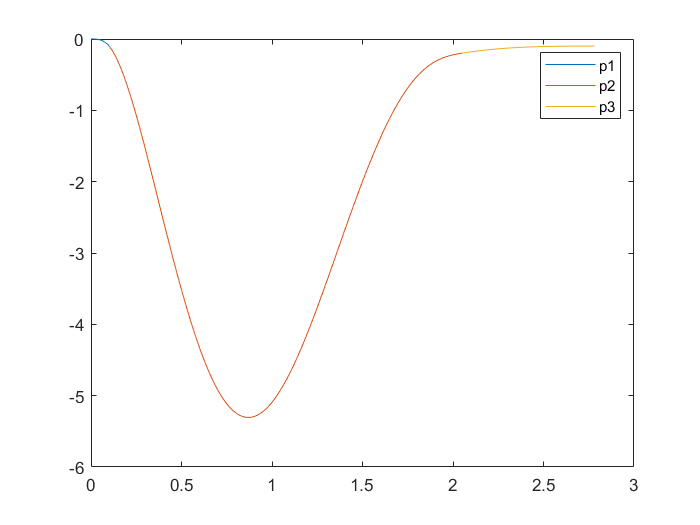


**(c)** Simulation Experiment 3

**(d)** Simulation Experiment 4

**Figure 5.** Optimal time trajectory planning position and pose of Seabuckthorn fruit vibrating manipulator X-axis


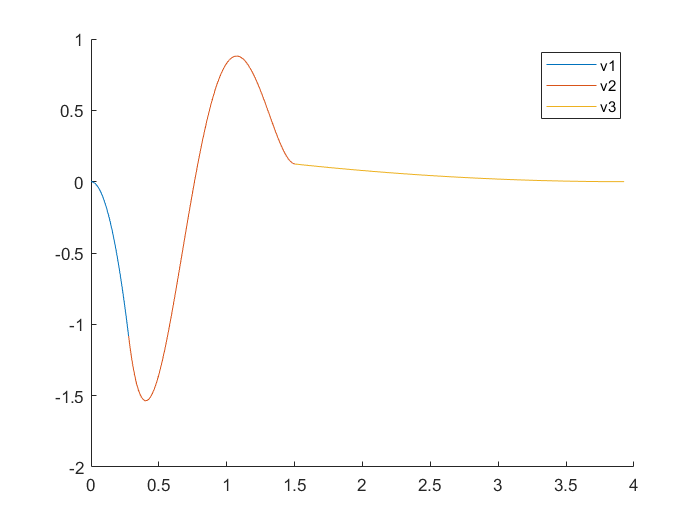

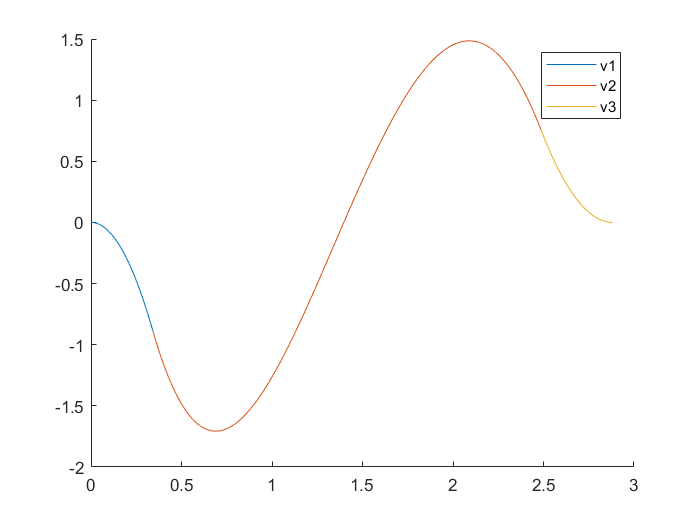


**(a)** Simulation Experiment 1

**(b)** Simulation Experiment 2


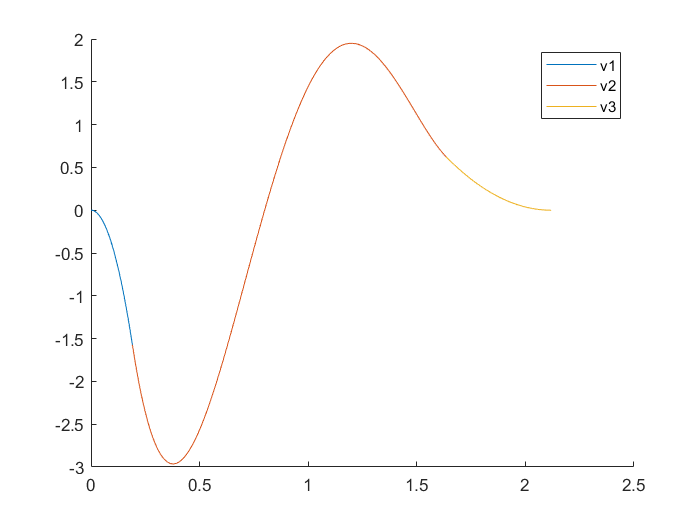

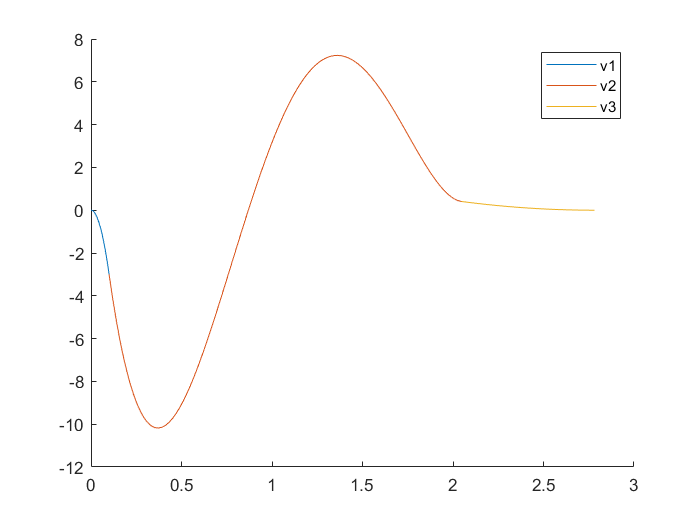


**(c)** Simulation Experiment 3

**(d)** Simulation Experiment 4

**Figure 6.** Simulation experiment of optimal time trajectory planning speed of manipulator X-axis


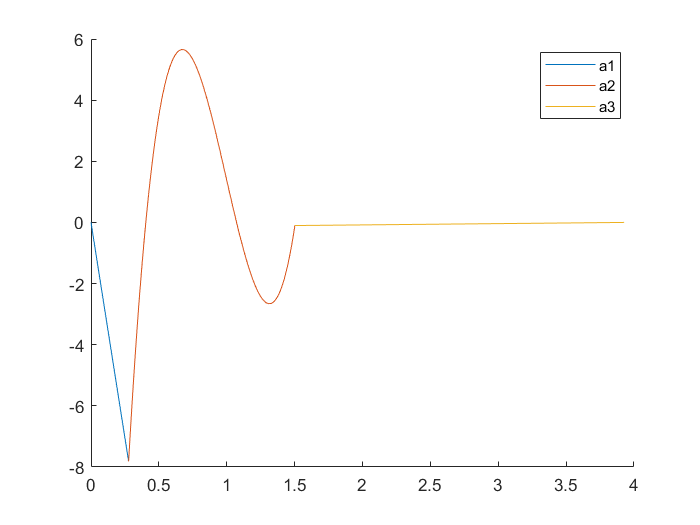

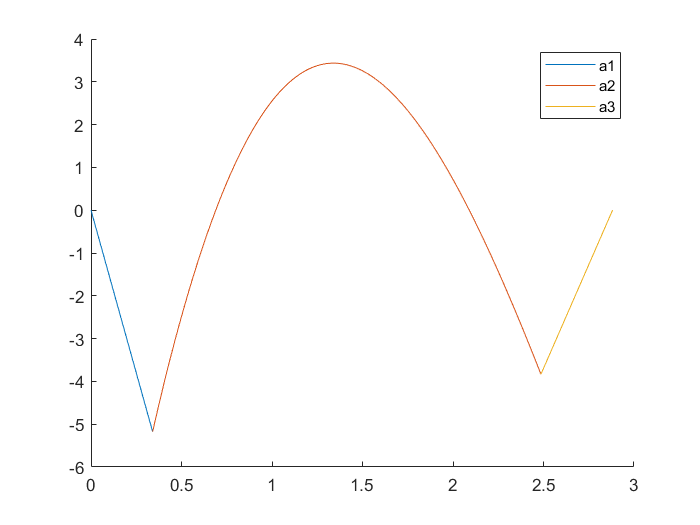


**(a)** Simulation Experiment 1

**(b)** Simulation Experiment 2


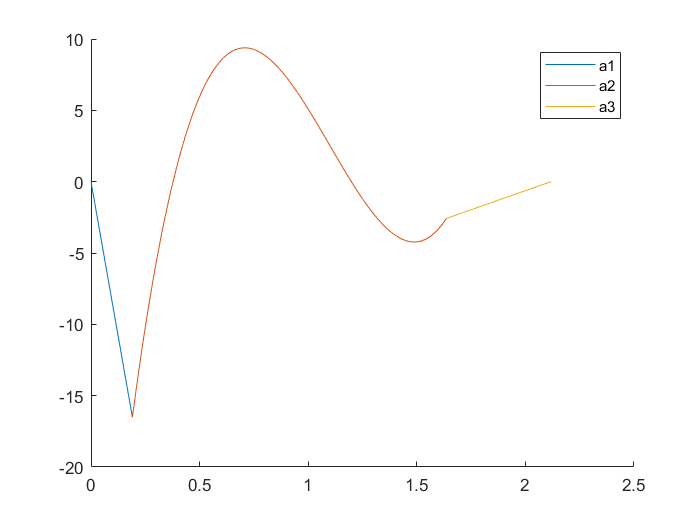

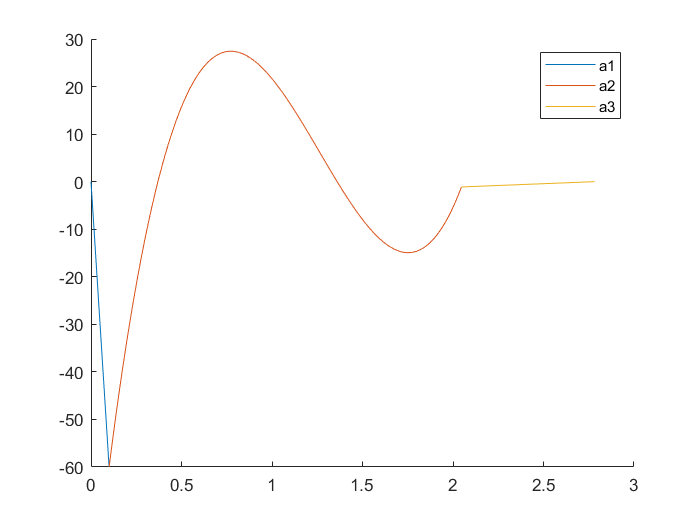


**(c)** Simulation Experiment 3

**(d)** Simulation Experiment 4

**Figure 7.** Acceleration simulation experiment of optimal time trajectory planning for X-axis manipulator


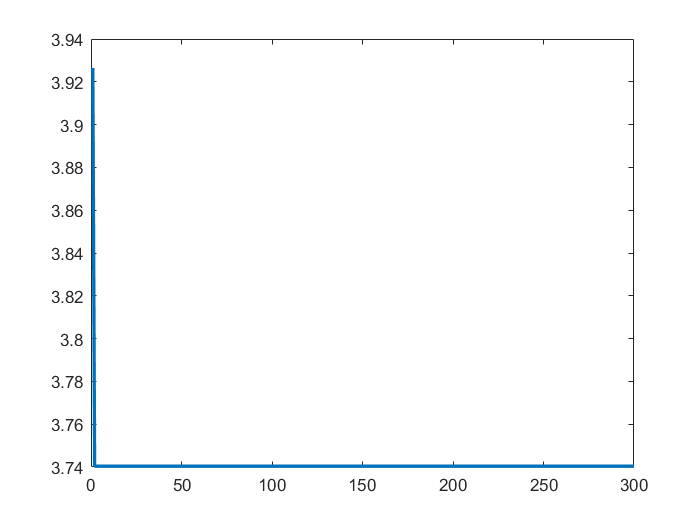

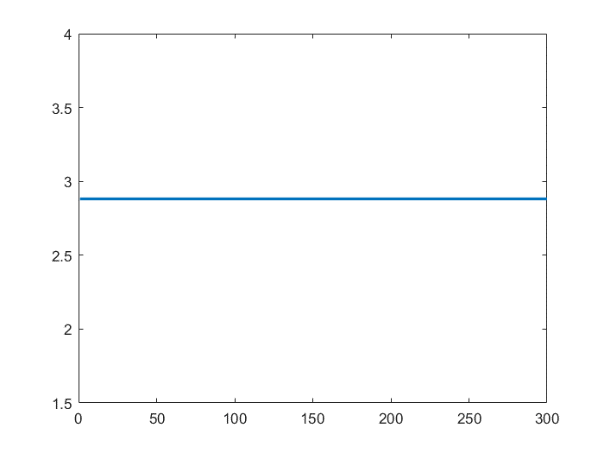


**(a)** Simulation Experiment 1

**(b)** Simulation Experiment 2


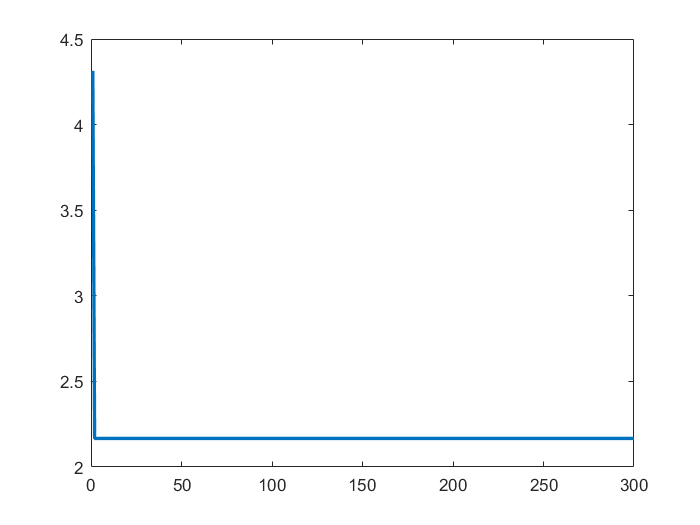

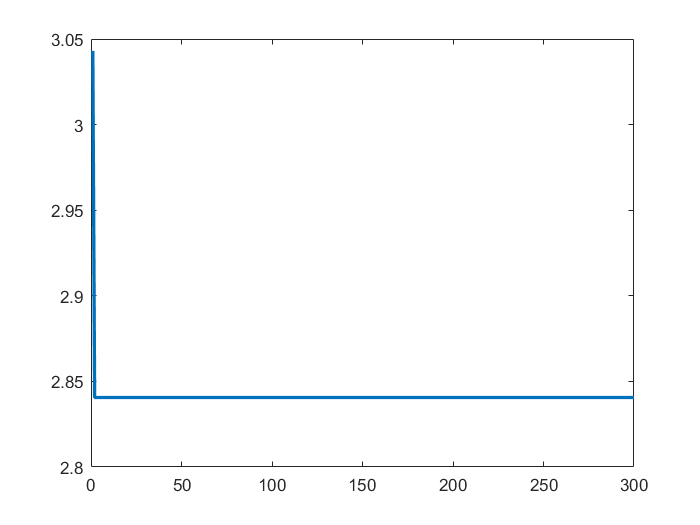


**(c)** Simulation Experiment 3

**(d)** Simulation Experiment 4

**Figure 8.** Simulation experiment on the applicability of the optimal time trajectory planning of the manipulator X-axis

**Table 3** Shows the experimental data of X-axis optimal time planning

| The first simulation experiment | | | | |
| --- | --- | --- | --- | --- |
| Global optimal position | 0.277066475400226 | 1.224813119188117 | 2.425329121475309 | 1.889139675298062 |
| Optimized  time | 2.425329121475309s | | | |
| The second simulation experiment | | | | |
| Global optimal position | 0.340507596131496 | 2.145354481328215 | 0.395649550235858 | 1.217984354310595 |
| Optimized  time | 0.395649550235858s | | | |
| The third simulation experiment | | | | |
| Global optimal position | 0.190642364061643 | 1.445231595233072 | 0.482514772124893 | 0.538512258125186 |
| Optimized  time | 0.482514772124893s | | | |
| The fourth simulation experiment | | | | |
| Global optimal position | 0.100000000000000 | 1. 946754616410645 | 0.735153935596233 | 1.386172223834769 |
| Optimized  time | 0.735153935596233s | | | |


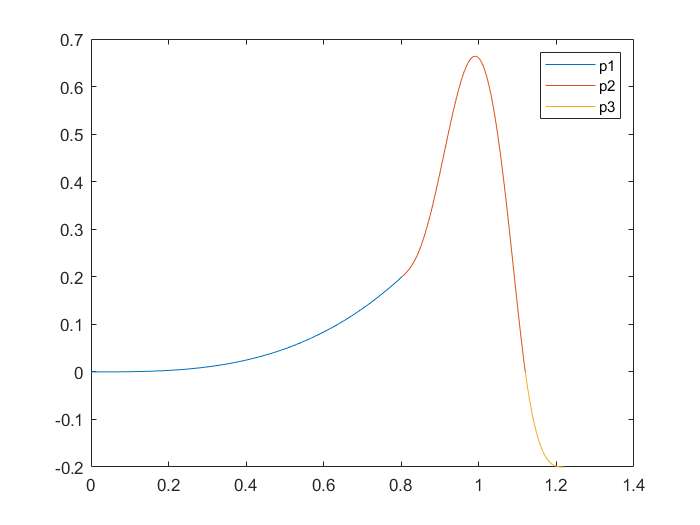

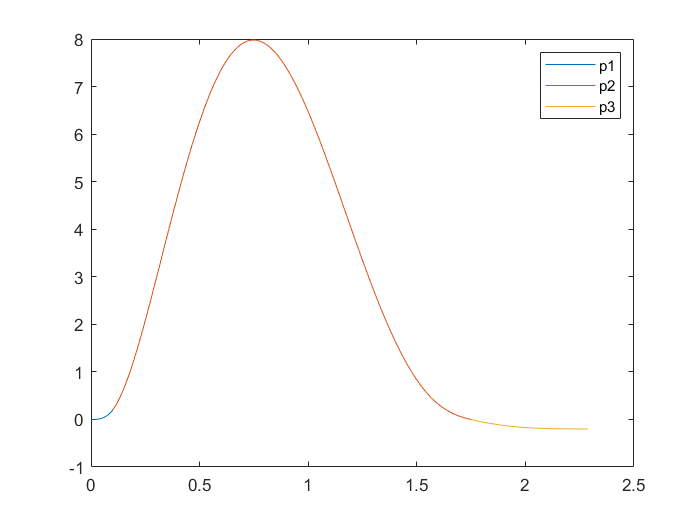


**(a)** Simulation Experiment 1

**(b)** Simulation Experiment 2


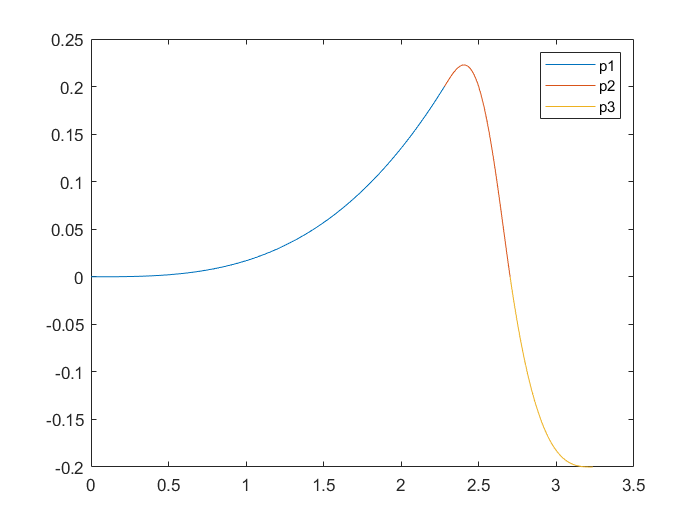


**(c)** Simulation Experiment 3

**Figure 9.** Simulation experiment of optimal time trajectory planning of manipulator Y-axis


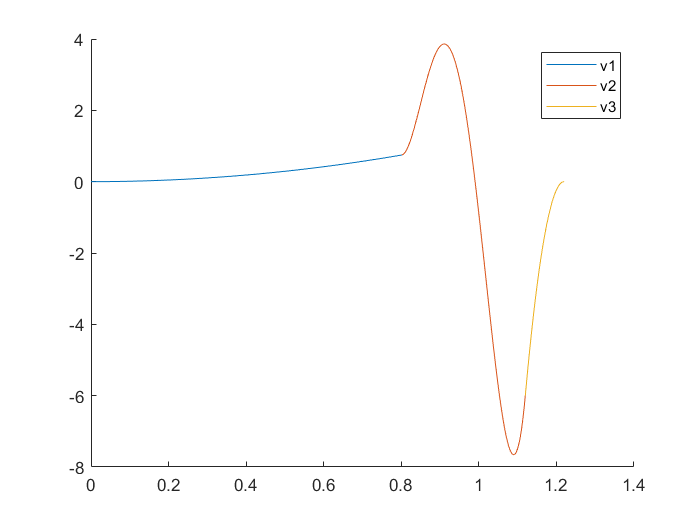

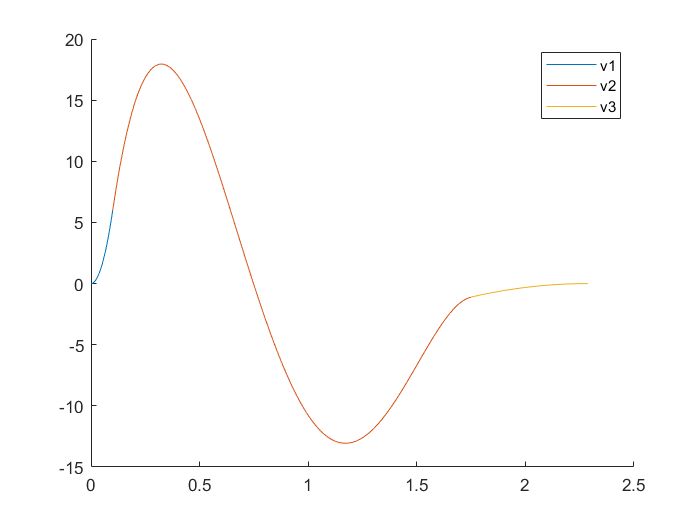


**(a)** Simulation Experiment 1

**(b)** Simulation Experiment 2


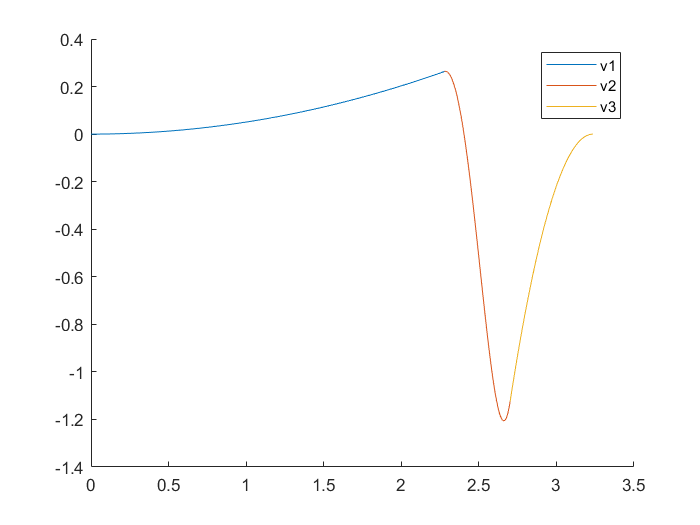


**(c)** Simulation Experiment 3

**Figure 10.** Simulation experiment of optimal time trajectory planning speed of manipulator Y-axis


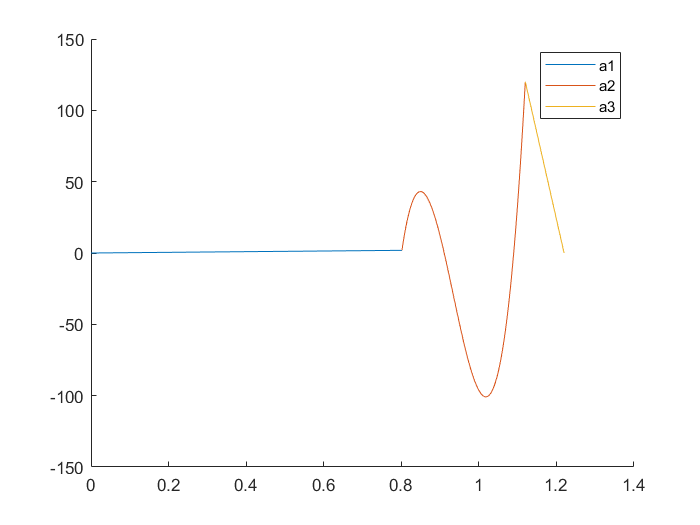

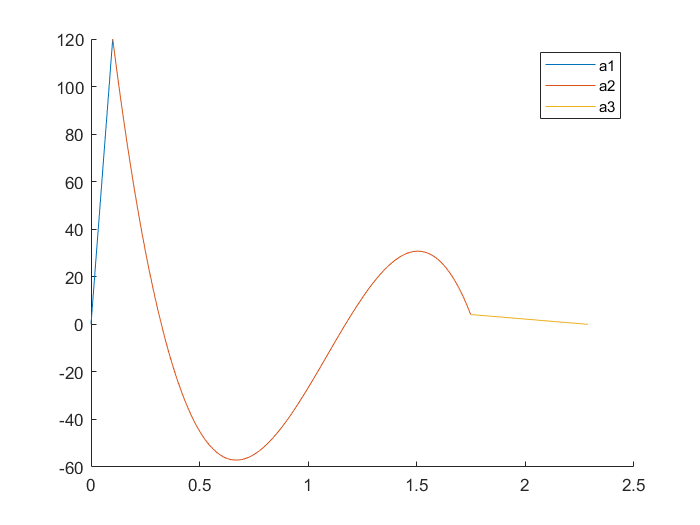


**(a)** Simulation Experiment 1

**(b)** Simulation Experiment 2


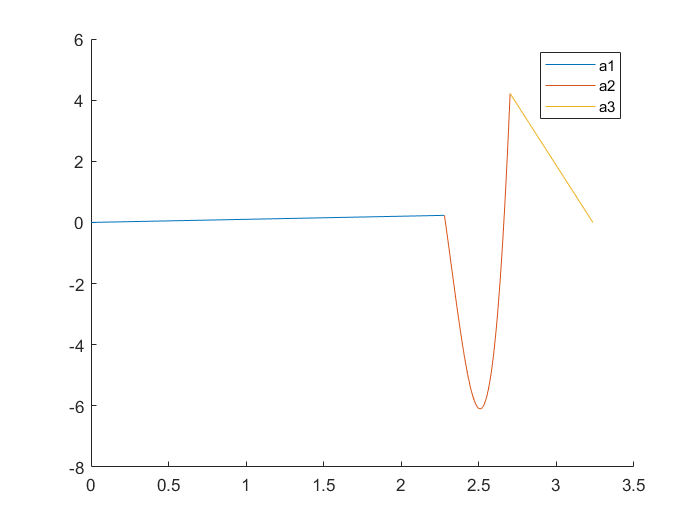


**(c)** Simulation Experiment 3

**Figure 11.** Acceleration simulation experiment of optimal time trajectory planning for Y-axis of manipulator


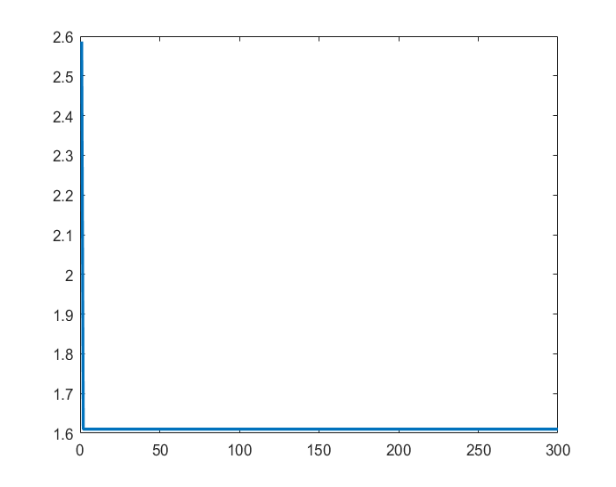

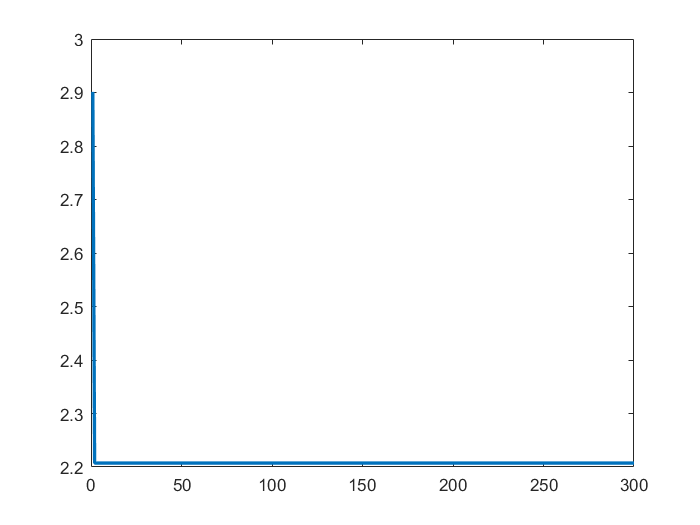


**(a)** Simulation Experiment 1

**(b)** Simulation Experiment 2


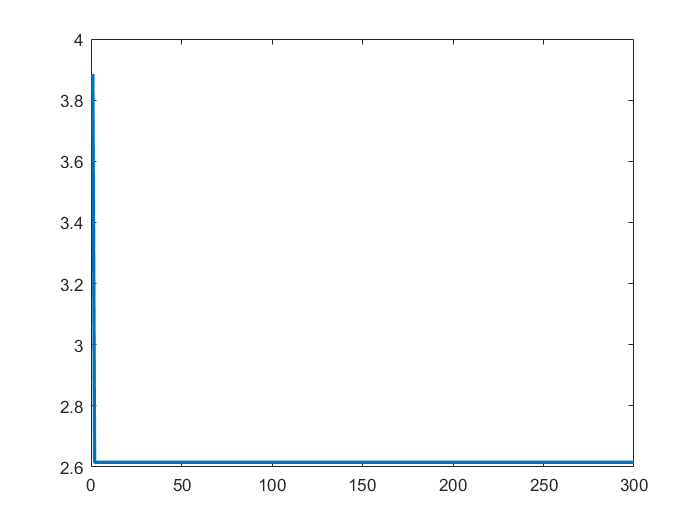


**(c)** Simulation Experiment 3

**Figure 12.** Simulation experiment of optimal time-path planning fitness of Y-axis of manipulator

**Table 4.** Shows the experimental data of Y-axis optimal time planning

| The first simulation experiment | | | | |
| --- | --- | --- | --- | --- |
| Global optimal position | 0.801727584194496 | 0.318015893327197 | 0.100000000000000 | 2.888148399733364 |
| Optimized  time | 0. 801727584194496s | | | |
| The second simulation experiment | | | | |
| Global optimal position | 0.100000000000000 | 1.648205608812784 | 0.5394 75779445781 | 1.241428451846682 |
| Optimized  time | 0.539475779445781s | | | |
| The third simulation experiment | | | | |
| Global optimal position | 2.278038103810259 | 0.423375252316917 | 0.533392454979409 | 2.810062021236965 |
| Optimized  time | 0.533392454979409s | | | |


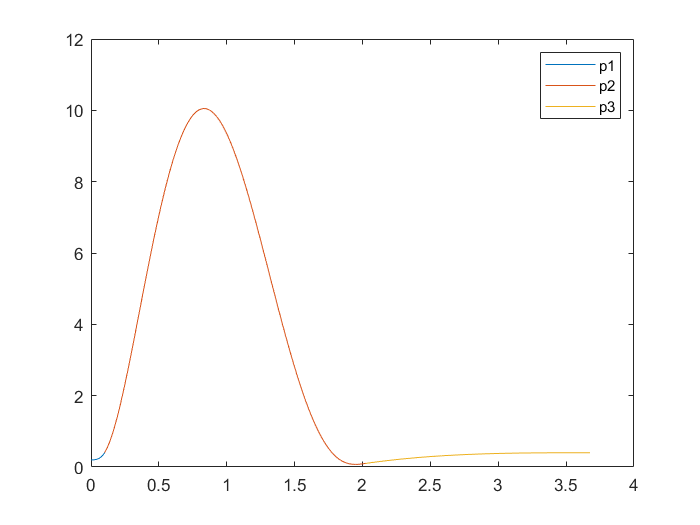

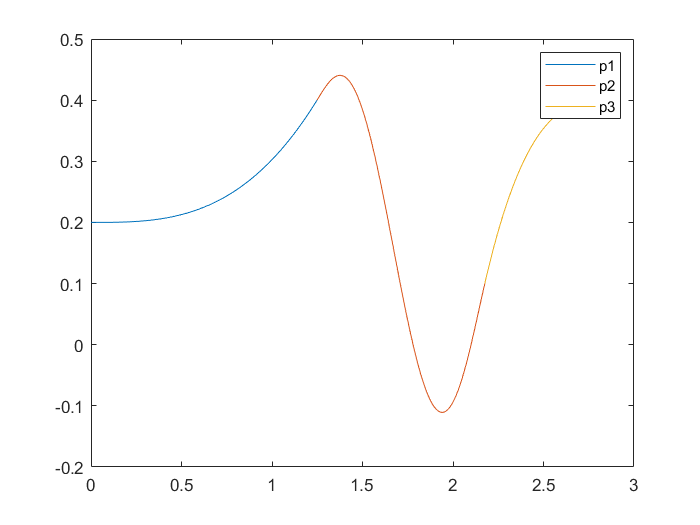


**(a)** Simulation Experiment 1

**(b)** Simulation Experiment 2


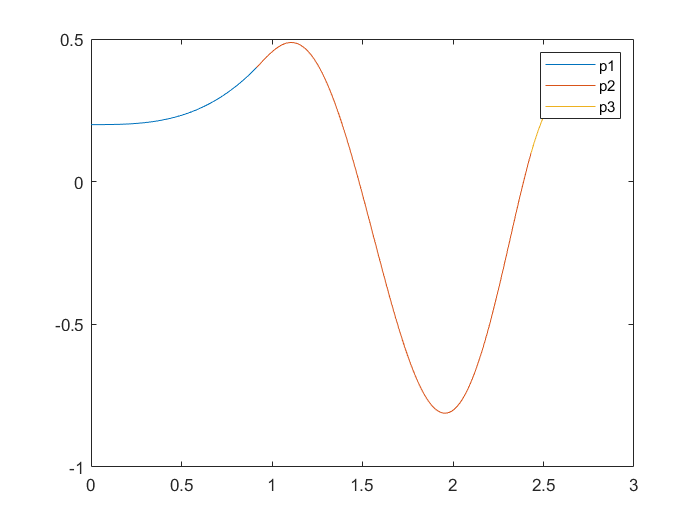

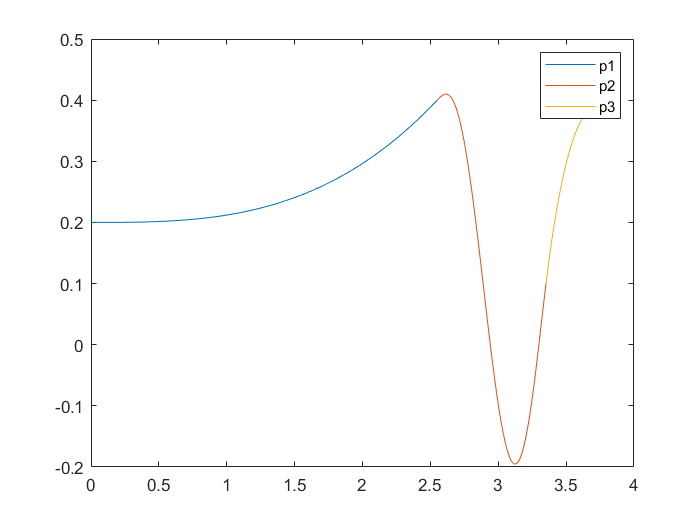


**(c)** Simulation Experiment 3

**(d)** Simulation Experiment 4

**Figure 13.** Simulation experiment of optimal time trajectory planning of Z-axis manipulator


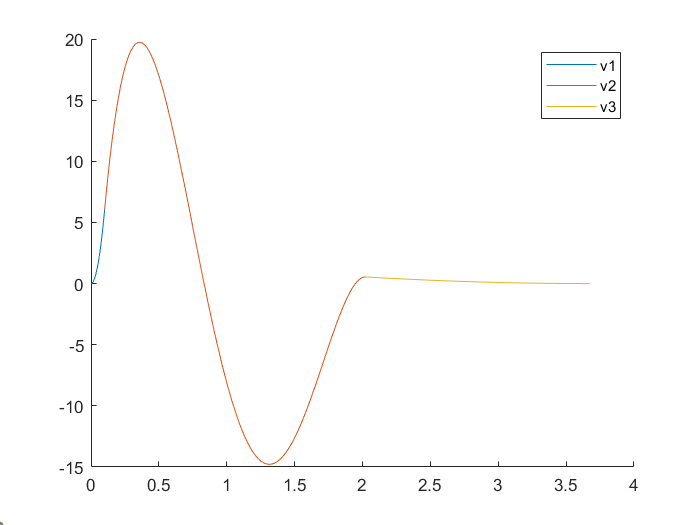

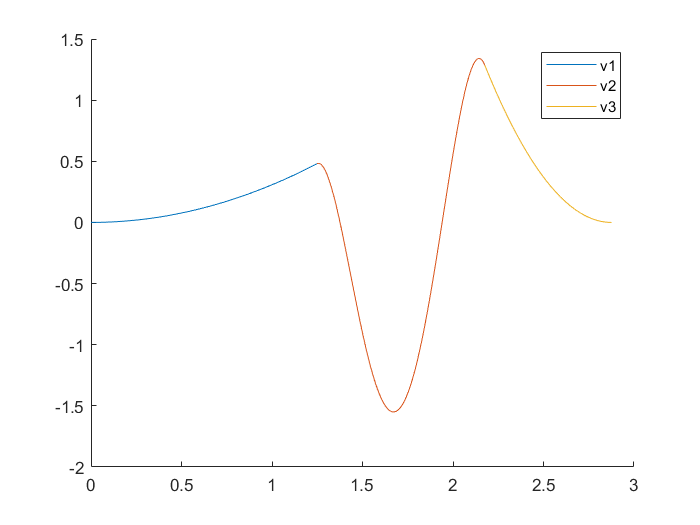


**(a)** Simulation Experiment 1

**(b)** Simulation Experiment 2


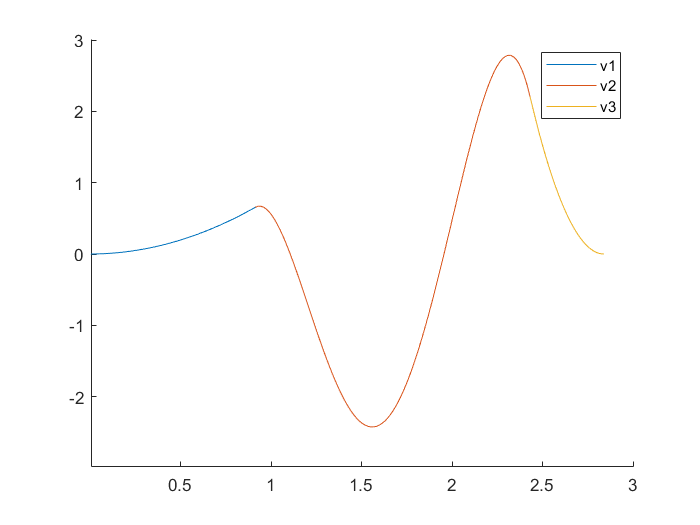

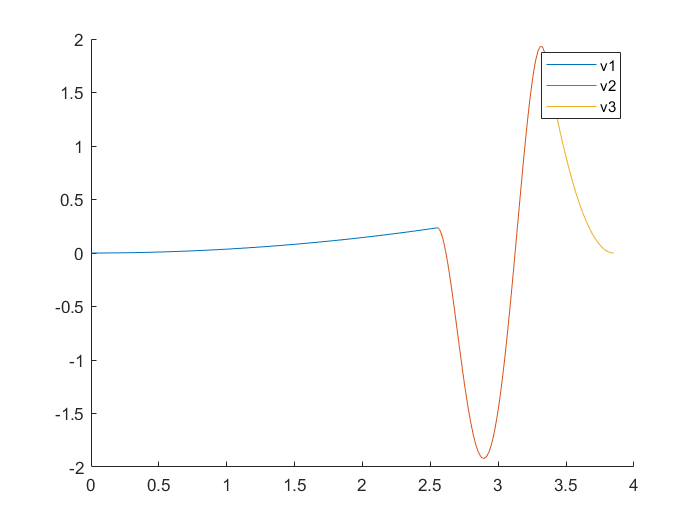


**(c)** Simulation Experiment 3

**(d)** Simulation Experiment 4

**Figure 14.** Simulation experiment of optimal time trajectory planning speed of Z-axis manipulator


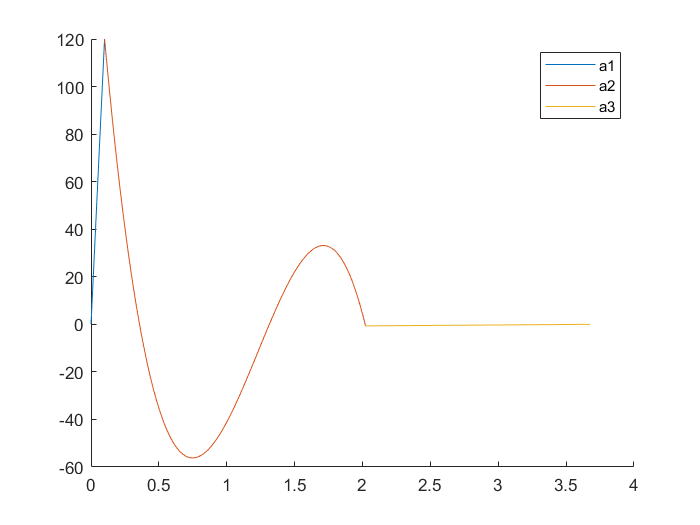

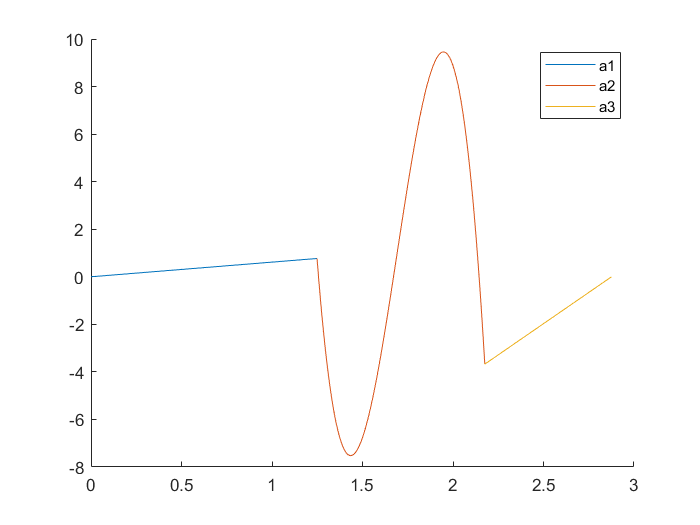


**(a)** Simulation Experiment 1

**(b)** Simulation Experiment 2


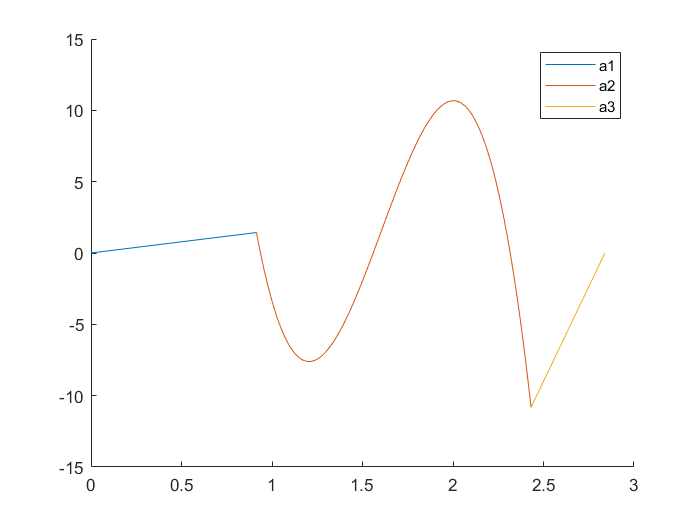

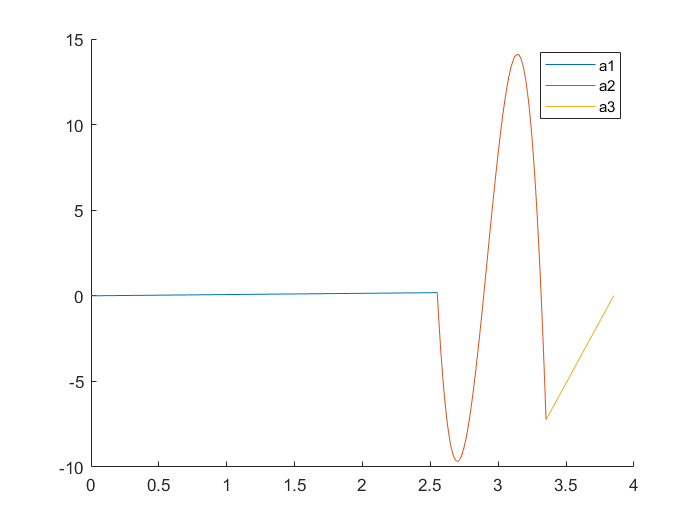


**(c)** Simulation Experiment 3

**(d)** Simulation Experiment 4

**Figure 15.** Acceleration simulation experiment of Z-axis optimal time trajectory planning of manipulator


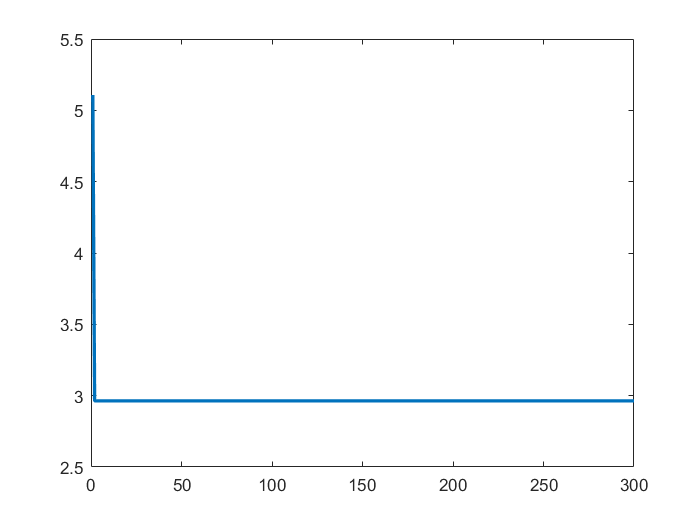

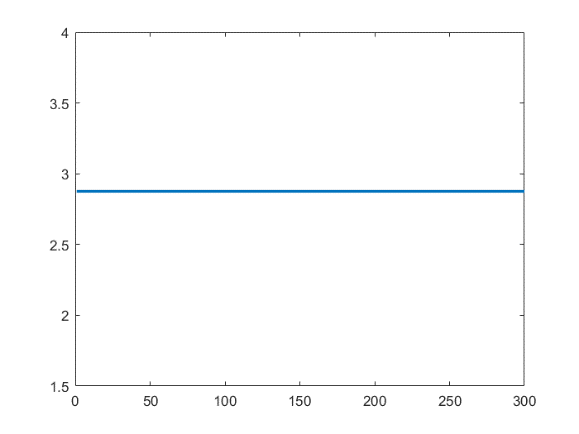


**(a)** Simulation Experiment 1

**(b)** Simulation Experiment 2


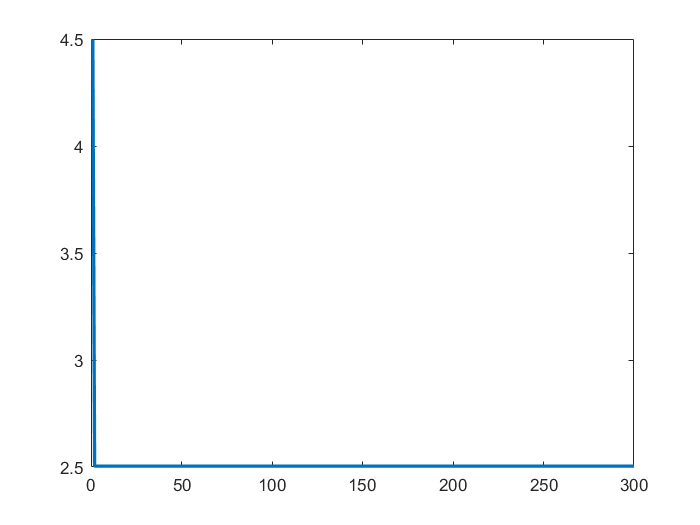

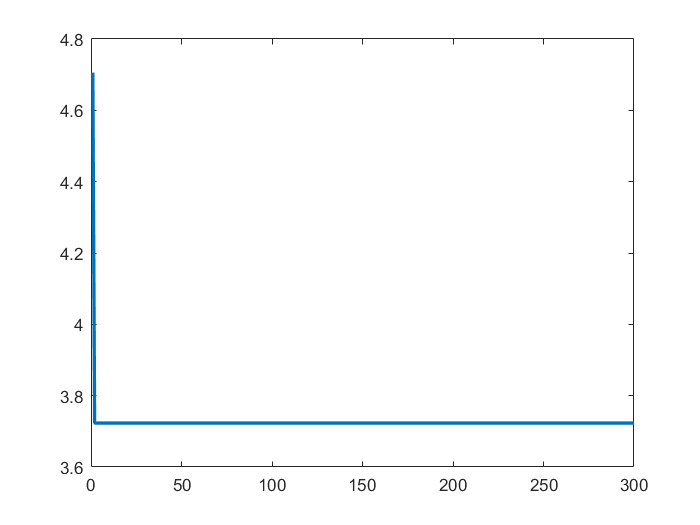


**(c)** Simulation Experiment 3

**(d)** Simulation Experiment 4

**Figure 16.** Simulation experiment on applicability of Z-axis optimal time trajectory planning of manipulator

**Table 5** Shows the experimental data of Z-axis optimal time planning

| The first simulation experiment | | | | |
| --- | --- | --- | --- | --- |
| Global optimal position | 0.100000000000000 | 1.922104073011805 | 1.653716843688830 | 0.331691192750019 |
| Optimized  time | 1.653716843688830s | | | |
| The second simulation experiment | | | | |
| Global optimal position | 1.248857358930636 | 0.926811517252962 | 0.699974684292500 | 2.167166739475434 |
| Optimized  time | 0.699974684292500s | | | |
| The third simulation experiment | | | | |
| Global optimal position | 0.914300333888886 | 1.516951342917716 | 0.407957540831187 | 1.760427957281867 |
| Optimized  time | 0.407957540831187s | | | |
| The fourth simulation experiment | | | | |
| Global optimal position | 2.550426624441166 | 0.801556750142424 | 0.498299013057836 | 0.865455540927244 |
| Optimized  time | 0.498299013057836s | | | |

**4. Experiment and analysis**

*Experimental purposes:*

To verify the effectiveness of the algorithm, a series of experiments are carried out, and the experimental results are analyzed.

*Materials and methods:*

The 2022 were collected from the 9th Division Hippophae rhamnoides planting base of Xinjiang Production and Construction Corps in October, three-year-old and four-year-old groups were placed in the refrigerator, frozen for 3D, as shown in Figure 17. Because the greater the angle between the stem of Hippophae rhamnoides L. and the fruit pedicle axis, the smaller the fruit separation force, the force direction should be parallel to the fruit pedicle axis, as shown in Figure 18. The physical properties of Hippophae rhamnoides L. Fruit were shown in Table 6 when 0 ° C was added to the experimental data.


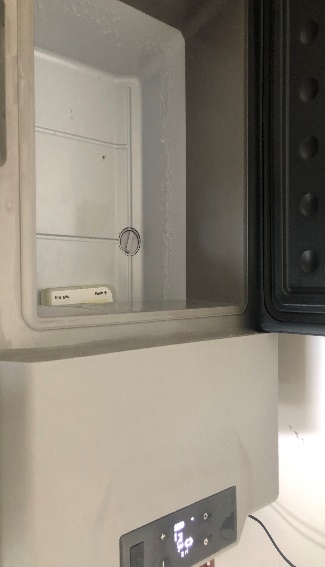

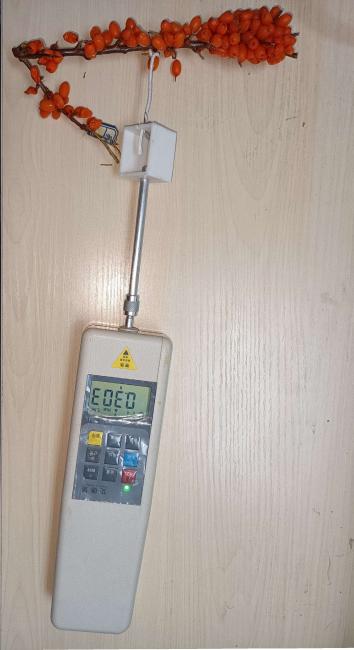


**Figure 17.** Refrigeration unit **Figure 18.** Force measuring device

**Table 6.** Physical characteristics of Hippophae rhamnoides L

| Tree age | Three years old | | | Four years old | | |
| --- | --- | --- | --- | --- | --- | --- |
| Frozen storage temperature/℃ | -18 | 0 | 3 | -18 | 0 | 3 |
| Fruit stalk diameter/mm | 0.66±0.14a | 0.65±0.23a | 0.65±0.23a | 0.70±0.05b | 0.72±0.14b | 0.72±0.14b |
| Fruit diameter/mm | 6.73±0.39a | 6.61±0.43a | 6.60±0.39a | 7.65±0.46b | 7.28±0.46b | 7.24±0.48b |
| Fruit longitudinal diameter/mm | 11.65±0.60a | 11.50±0.59a | 11.49±0.60a | 12.17±0.59b | 12.13±0.58b | 12.13±0.57b |
| Fruit quality/g | 0.35±0.04a | 0.35±0.04a | 0.35±0.04a | 0.57±0.05b | 0.52±0.08b | 0.51±0.08b |
| Pericarp rupture load/g | 164.32±15.81a | 118.50±8.81a | 110.86±7.75b | 165.25±9.83a | 120.37±5.83a | 112.89±5.25b |
| Pericarp hardness/(g/mm^2^) | 52.33±3.4a | 37.73±3.40a | 35.30±2.93b | 52.63±3.5a | 38.33±2.17a | 35.95±1.98b |
| Separation force of fruit/N | 1.18±0.16a | 1.10±0.21a | 1.09±0.20a | 1.79±0.29b | 1.75±0.24b | 1.74±0.23b |

Note: different lowercase letters after peer-to-peer data showed significant difference at P < 0.05 level

Correlation Analysis of physical characteristics of Hippophae rhamnoides L. branches and fruits:

Spearman correlation analysis is a non-parametric statistical method used to evaluate the relationship between two variables. It is based on Spearman's rank correlation coefficient, which measures the monotonic relationship between two variables (whether the other variable increases or decreases as one variable increases). The Spearman correlation analysis is suitable for cases where the data do not conform to the normal distribution or there are outliers. It can evaluate linear relationships as well as non-linear relationships.

Spearman correlation analysis using SPSS Software was selected for this study, as shown in Table 7.

**Table 7.** Spearman correlation-standard format

| Spearman correlation | Average | Standard deviation | Tree age | Frozen storage temperature/℃ | Separation force of fruit/N | Fruit stalk diameter/mm | Fruit diameter/mm | Fruit longitudinal diameter/mm | Fruit quality/g | Pericarp rupture load/g | Pericarp hardness/(g/mm^2^) |
| --- | --- | --- | --- | --- | --- | --- | --- | --- | --- | --- | --- |
| Tree age | 1.5 | 0.548 | 1 |  |  |  |  |  |  |  |  |
| Frozen storage  temperature/℃ | 2 | 0.894 | 0 | 1 |  |  |  |  |  |  |  |
| Separation force of fruit/N | 3.5 | 1.871 | 0.878* | -0.478 | 1 |  |  |  |  |  |  |
| Fruit stalk diameter/mm | 2.5 | 1.378 | 0.905* | 0 | 0.794 | 1 |  |  |  |  |  |
| Fruit diameter/mm | 3.5 | 1.871 | 0.878* | -0.478 | 1.000** | 0.794 | 1 |  |  |  |  |
| Fruit longitudinal diameter/mm | 3.5 | 1.871 | 0.878* | -0.478 | 1.000** | 0.794 | 1.000** | 1 |  |  |  |
| Fruit quality/g | 2 | 1.265 | 0.933** | -0.254 | 0.941** | 0.75 | 0.941** | 0.941** | 1 |  |  |
| Pericarp rupture load/g | 3.5 | 1.871 | 0.293 | -0.956** | 0.714 | 0.265 | 0.714 | 0.714 | 0.516 | 1 |  |
| Pericarp hardness/(g/mm^2^) | 3.5 | 1.871 | 0.293 | -0.956** | 0.714 | 0.265 | 0.714 | 0.714 | 0.516 | 1.000** | 1 |
| * p<0.05 ** p<0.01 | | | | | | | | | | | |

From Table 7, the relationship between tree age and fruit separation force, fruit stalk diameter, fruit transverse diameter, fruit longitudinal diameter, and fruit quality were all significant. The correlation coefficients were 0.878,0.905,0.878,0.878,0.933, respectively, all of them were greater than 0, which meant that there was a positive correlation between tree age and fruit separation force, fruit stalk diameter, fruit transverse diameter, fruit longitudinal diameter and fruit quality. At the same time, there was no significant relationship between tree age and freezing storage temperature, Peel hardness under a load of cracking, and the correlation coefficient was close to 0, there was no correlation between the 3 items of pericarp hardness.

*Test factors:*

Through Spearman correlation analysis, the four-year-old “Late autumn red” trees were selected for this study, and all of them were vibrating at the same temperature and the same location on the same vibration test stand, therefore, no need to consider the tree age, species, and vibration source location factors. Finally, combined with the planning of vibration trajectory and the study of the law of fruit shedding, the following three factors were selected for the orthogonal experiment: frequency, amplitude, and vibration time, the influence of the planned vibration trajectory on the deactivation effect is studied. This is shown in Table 8.

**Table 8.** Test level table

| Horizontal serial number | Vibration frequency/Hz | Amplitude of vibration/mm | Vibration time/s |
| --- | --- | --- | --- |
| Level 1 | 21.1 | 30 | 2 |
| Level 2 | 16.2 | 30 | 4 |
| Level 3 | 14.4 | 20 | 4 |

An orthogonal table is a set of regular design tables, denoted as L_n_ (t ^c^). L is the number of orthogonal tables, n is the number of experiments, t is the level number, and c is the sequence number. In this study, the orthogonal test is three factors and three levels, so the orthogonal test scheme table is designed as Table 9.

**Table 9.** Orthogonal experimental design table

| No | Vibration frequency/Hz | Amplitude of vibration/mm | Vibration time/s | ys5 |
| --- | --- | --- | --- | --- |
| 1 | 21.1 | 30 | 0.2 | 1 |
| 2 | 21.1 | 30 | 0.4 | 3 |
| 3 | 21.1 | 20 | 0.4 | 2 |
| 4 | 16.2 | 30 | 0.4 | 3 |
| 5 | 16.2 | 30 | 0.4 | 2 |
| 6 | 16.2 | 20 | 0.2 | 1 |
| 7 | 14.4 | 30 | 0.4 | 2 |
| 8 | 14.4 | 30 | 0.2 | 1 |
| *9* | *14.4* | *20* | *0.4* | *3* |

*One-way orthogonal test analysis of variance:*

Analysis of variance of fruit drop rate:

Using SPSS software to substitute the orthogonal experimental data for one-way analysis of variance, Table 10 ~ 11.

**Table 10.** Inter-body effect test of fruit drop rate

| Source | Class III sum of squares | Degree of freedom | Mean Square | F | P |
| --- | --- | --- | --- | --- | --- |
| Modify the model | 0.003^a^ | 7 | 0 | 159.763 | 0.061 |
| Intercept | 7.029 | 1 | 7.029 | 3135621.505 | 0 |
| Vibration frequency/Hz | 0.002 | 2 | 0.001 | 413.748 | 0.035 |
| Amplitude of vibration/mm | 0 | 1 | 0 | 155.711 | 0.051 |
| Vibration time/s | 0 | 1 | 0 | 58.787 | 0.083 |
| Vibration frequency/Hz * Amplitude of vibration/mm | 7.12E-05 | 1 | 7.12E-05 | 31.781 | 0.112 |
| Vibration frequency/Hz * Vibration time/s | 1.13E-06 | 1 | 1.13E-06 | 0.505 | 0.607 |
| Amplitude of vibration/mm * Vibration time/s | 0 | 0 | . | . | . |
| Vibration frequency/Hz * Amplitude of vibration/mm * Vibration time/s | 0 | 0 | . | . | . |
| Error | 2.24E-06 | 1 | 2.24E-06 |  |  |
| Total | 8.33 | 9 |  |  |  |
| Corrected total | 0.003 | 8 |  |  |  |

a. R Square=0.999(Adjusted R Square=0.993)

In Table 10, the significance of the modified model is greater than 0.05, so there is no significant difference between the modified model and the whole analysis of variance model, and the effect is vibration frequency > vibration amplitude > vibration time.

A post-test analysis was performed for the frequencies with higher impact factors using the least significant difference (LSD) method with one of the vibration frequencies and the other two references, as shown in Table 11.

**Table 11.** LSD post hoc analysis of fruit drop rate

| Method | (I) Vibration frequency/Hz | (J) Vibration frequency/Hz | Mean difference (I-J) | Standard error | P | 95% Confidence interval | |
| --- | --- | --- | --- | --- | --- | --- | --- |
|  |  |  |  |  |  | The lower limit | Ceiling |
| LSD | 14.4 | 16.2 | 1.43% | 0.12% | 0.054 | -2.99% | 0.12% |
|  |  | 21.1 | 3.65%^*^ | 0.12% | 0.021 | -5.20% | -2.10% |
|  | 16.2 | 14.4 | 1.44% | 0.12% | 0.054 | -0.12% | 2.99% |
|  |  | 21.1 | -2.21%^*^ | 0.12% | 0.035 | -3.77% | -0.66% |
|  | 21.1 | 14.4 | 3.65%^*^ | 0.12% | 0.021 | 2.10% | 5.20% |
|  |  | 16.2 | 2.21%^*^ | 0.12% | 0.035 | 0.66% | 3.77% |

Based on the measured average.

The error term is the mean square (error) =0.000002242.

*. The significance level of mean difference was 0.05.

According to Table 11, the significance was greater than 0.05 after only 14.4 Hz and 16.2 Hz, so only 14.4 Hz and 16.2 Hz were not different.

Residual plots were drawn to observe the effect of data fitting on fruit drop rates, as shown in Figure 19.


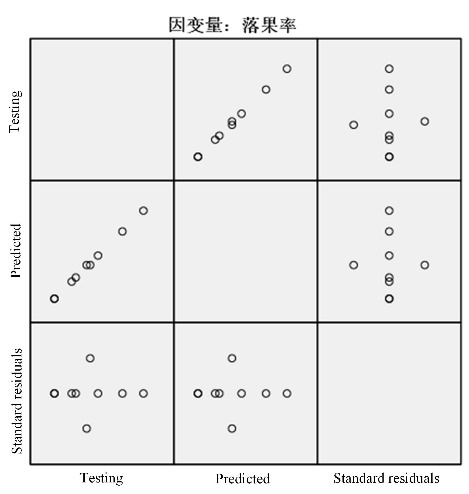


**Figure 19.** Standard residual diagram of fruit drop rate

According to Figure 19, the residual map is composed of predicted value, measured value and standard residual value, and the predicted value of fruit drop rate is obviously correlated with measured value.
