# Supplementary figures and images for: Trajectory analysis and optimization of sea buckthorn fruit vibration separation manipulator based on I-PSO algorithm
Source: Sci Rep. 2023 Nov 17;13:20124. doi: 10.1038/s41598-023-47001-2 (PMC10656555; doi:10.1038/s41598-023-47001-2)

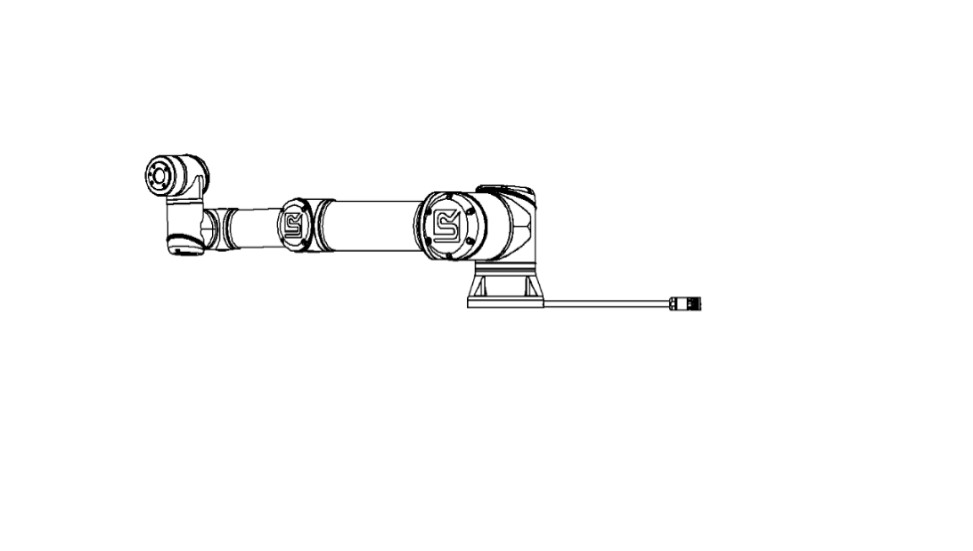

Supplement: Supplementary file 1 — Supplementary Figures. [file 41598_2023_47001_MOESM1_ESM.zip › Supplementary Figures/Figure 1. Schematic diagram of the robot arm.jpg]

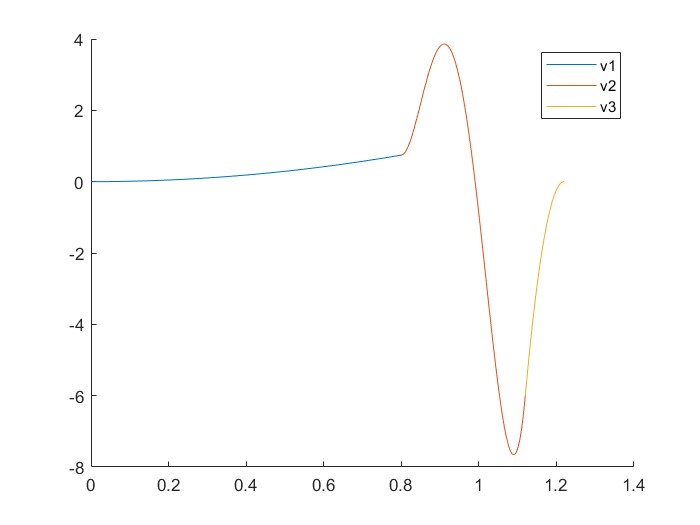

Supplement: Supplementary file 1 — Supplementary Figures. [file 41598_2023_47001_MOESM1_ESM.zip › Supplementary Figures/Figure 10.(a)Simulation Experiment 1.jpg]

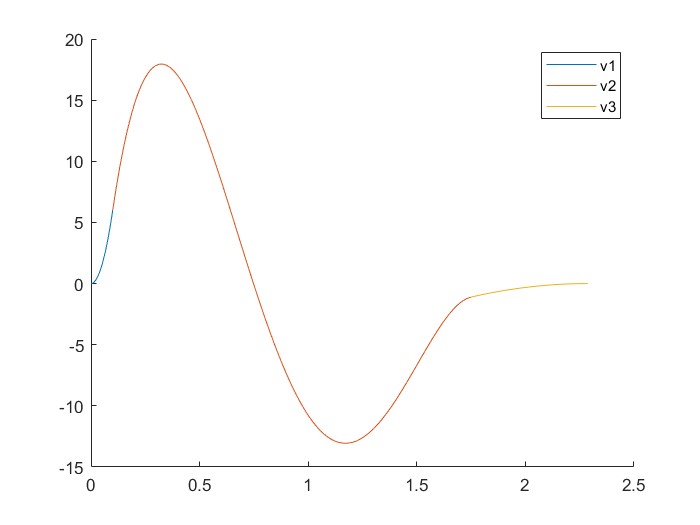

Supplement: Supplementary file 1 — Supplementary Figures. [file 41598_2023_47001_MOESM1_ESM.zip › Supplementary Figures/Figure 10.(b)Simulation Experiment 2.jpg]

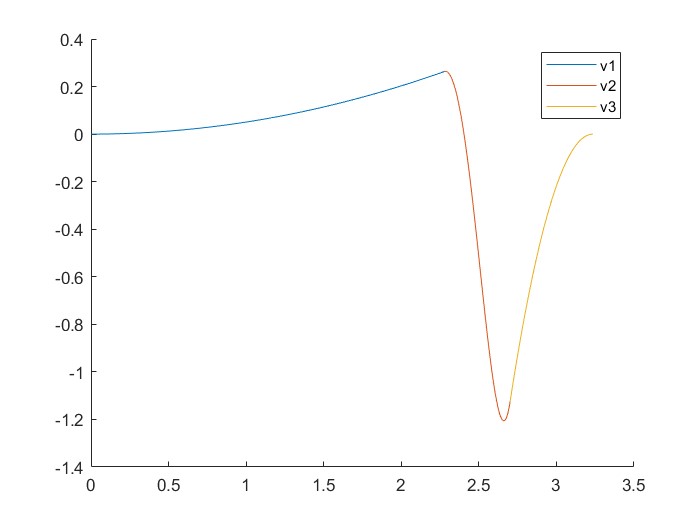

Supplement: Supplementary file 1 — Supplementary Figures. [file 41598_2023_47001_MOESM1_ESM.zip › Supplementary Figures/Figure 10.(c)Simulation Experiment 3.jpg]

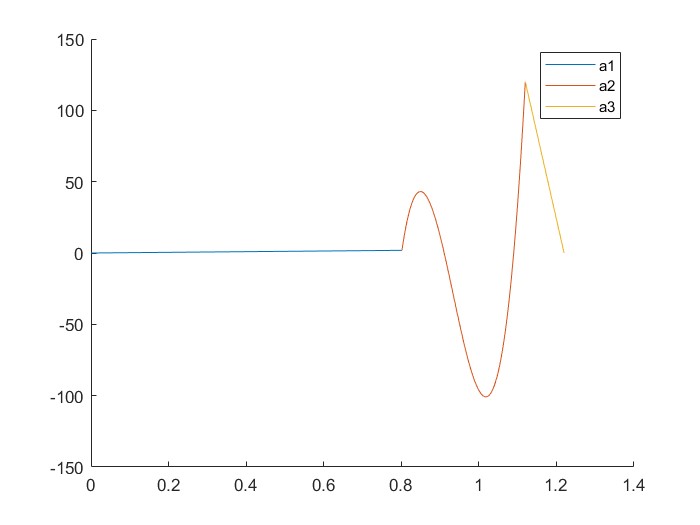

Supplement: Supplementary file 1 — Supplementary Figures. [file 41598_2023_47001_MOESM1_ESM.zip › Supplementary Figures/Figure 11.(a)Simulation Experiment 1.jpg]

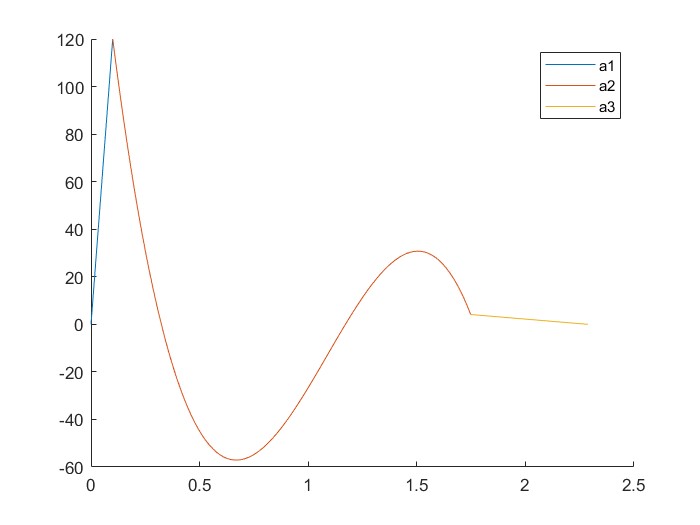

Supplement: Supplementary file 1 — Supplementary Figures. [file 41598_2023_47001_MOESM1_ESM.zip › Supplementary Figures/Figure 11.(b)Simulation Experiment 2.jpg]

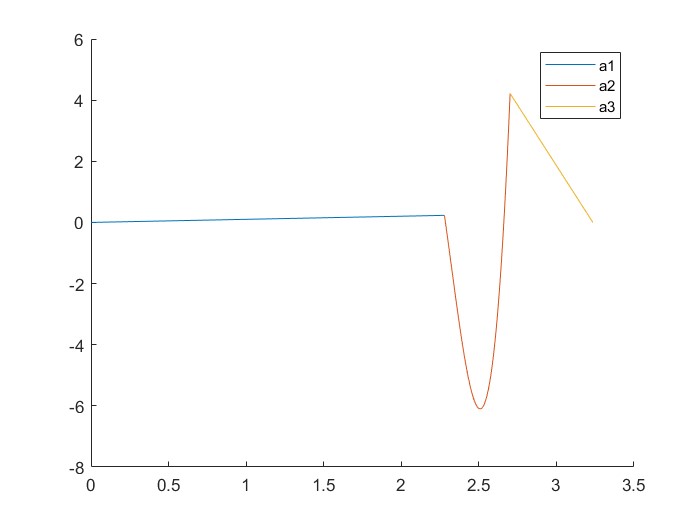

Supplement: Supplementary file 1 — Supplementary Figures. [file 41598_2023_47001_MOESM1_ESM.zip › Supplementary Figures/Figure 11.(c)Simulation Experiment 3.jpg]

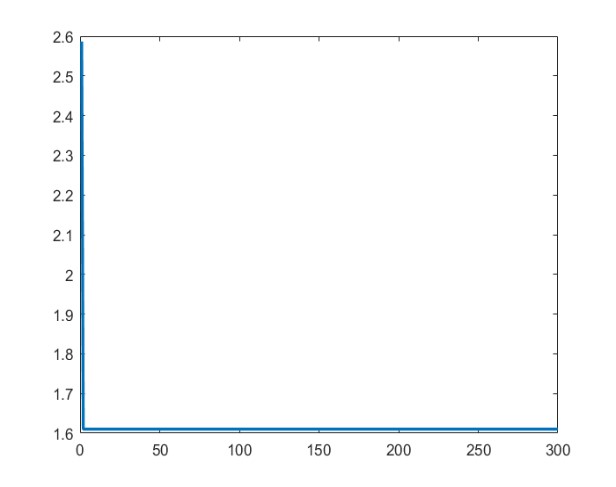

Supplement: Supplementary file 1 — Supplementary Figures. [file 41598_2023_47001_MOESM1_ESM.zip › Supplementary Figures/Figure 12.(a)Simulation Experiment 1.jpg]

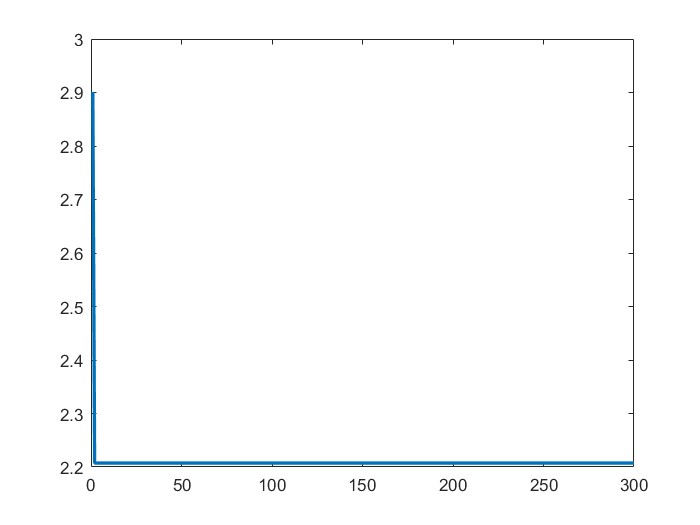

Supplement: Supplementary file 1 — Supplementary Figures. [file 41598_2023_47001_MOESM1_ESM.zip › Supplementary Figures/Figure 12.(b)Simulation Experiment 2.jpg]

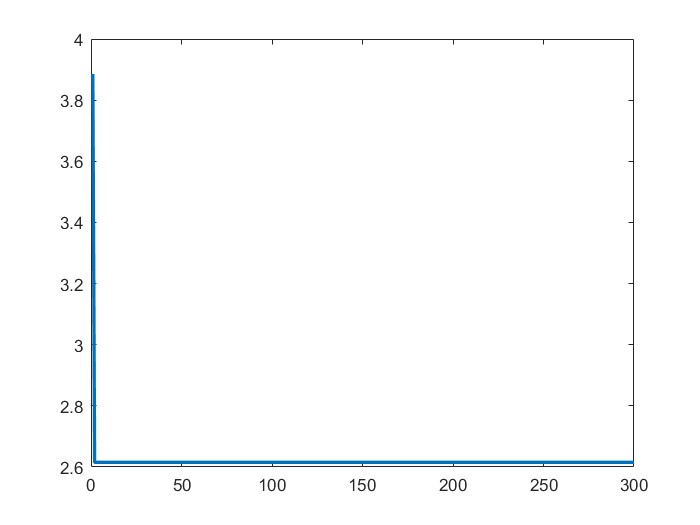

Supplement: Supplementary file 1 — Supplementary Figures. [file 41598_2023_47001_MOESM1_ESM.zip › Supplementary Figures/Figure 12.(c)Simulation Experiment 3.jpg]

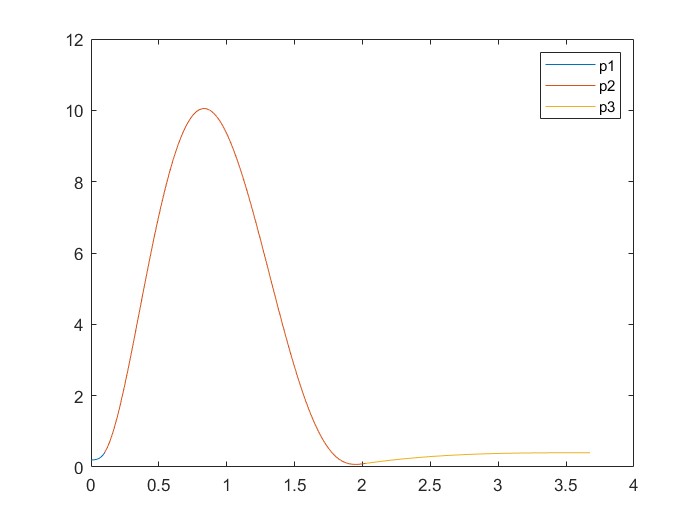

Supplement: Supplementary file 1 — Supplementary Figures. [file 41598_2023_47001_MOESM1_ESM.zip › Supplementary Figures/Figure 13.(a)Simulation Experiment 1.jpg]

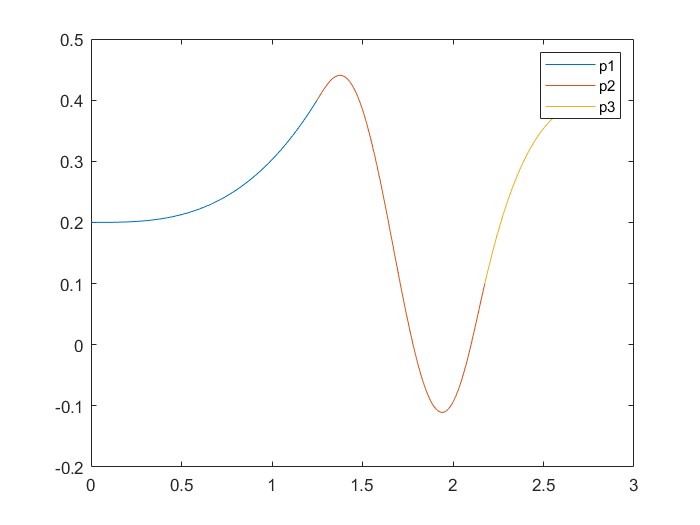

Supplement: Supplementary file 1 — Supplementary Figures. [file 41598_2023_47001_MOESM1_ESM.zip › Supplementary Figures/Figure 13.(b)Simulation Experiment 2.jpg]

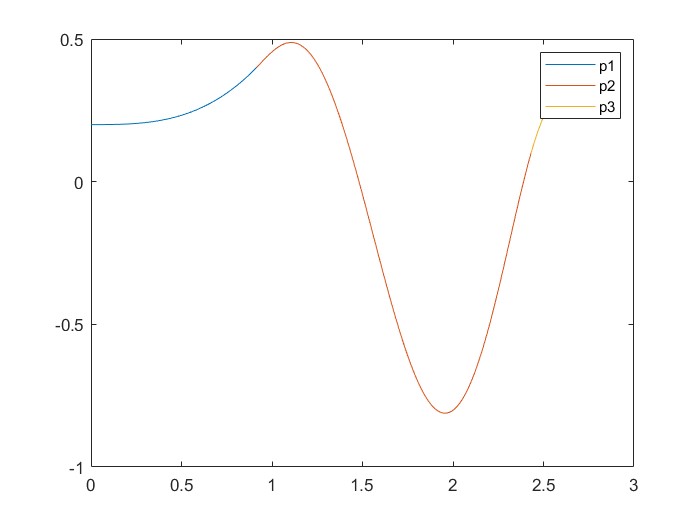

Supplement: Supplementary file 1 — Supplementary Figures. [file 41598_2023_47001_MOESM1_ESM.zip › Supplementary Figures/Figure 13.(c)Simulation Experiment 3.jpg]

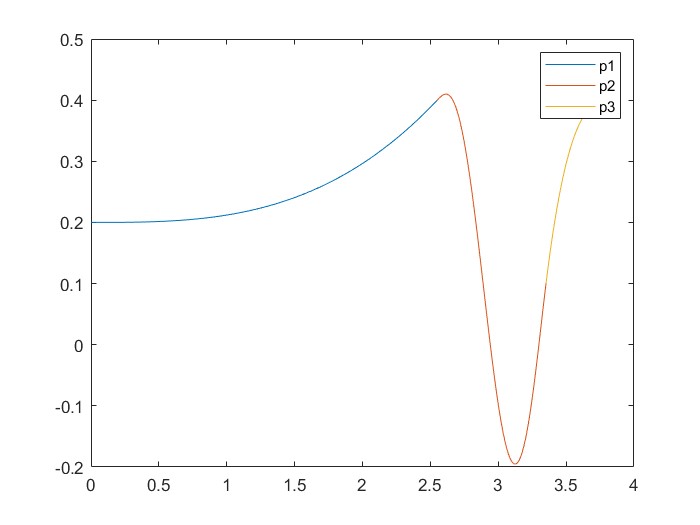

Supplement: Supplementary file 1 — Supplementary Figures. [file 41598_2023_47001_MOESM1_ESM.zip › Supplementary Figures/Figure 13.(d)Simulation Experiment 4.jpg]

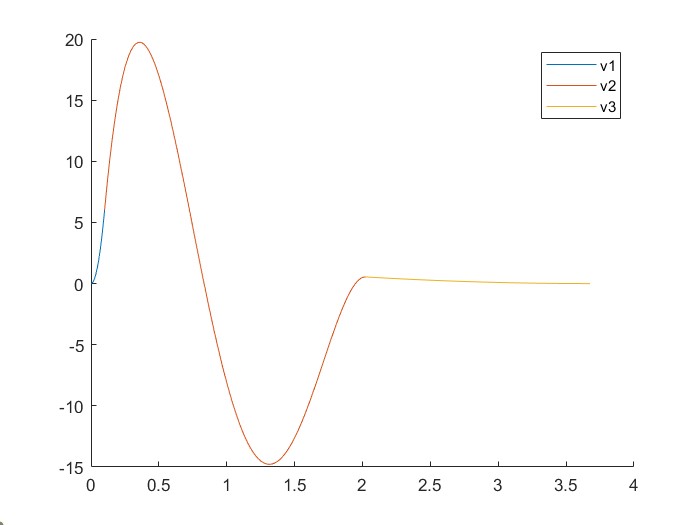

Supplement: Supplementary file 1 — Supplementary Figures. [file 41598_2023_47001_MOESM1_ESM.zip › Supplementary Figures/Figure 14.(a)Simulation Experiment 1.jpg]

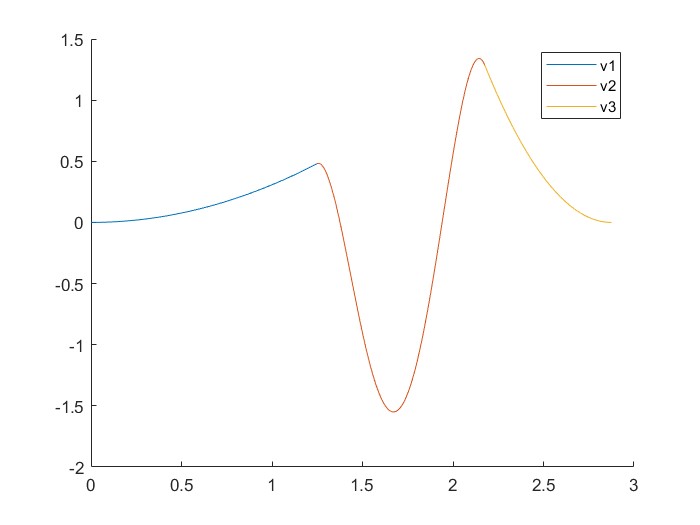

Supplement: Supplementary file 1 — Supplementary Figures. [file 41598_2023_47001_MOESM1_ESM.zip › Supplementary Figures/Figure 14.(b)Simulation Experiment 2.jpg]

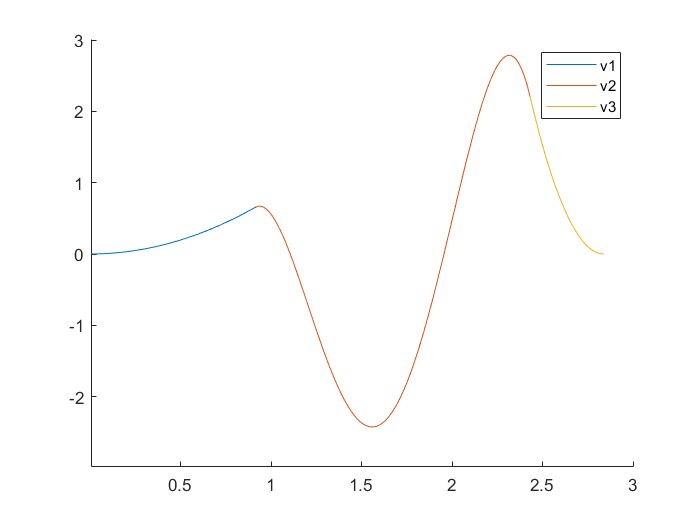

Supplement: Supplementary file 1 — Supplementary Figures. [file 41598_2023_47001_MOESM1_ESM.zip › Supplementary Figures/Figure 14.(c)Simulation Experiment 3.jpg]

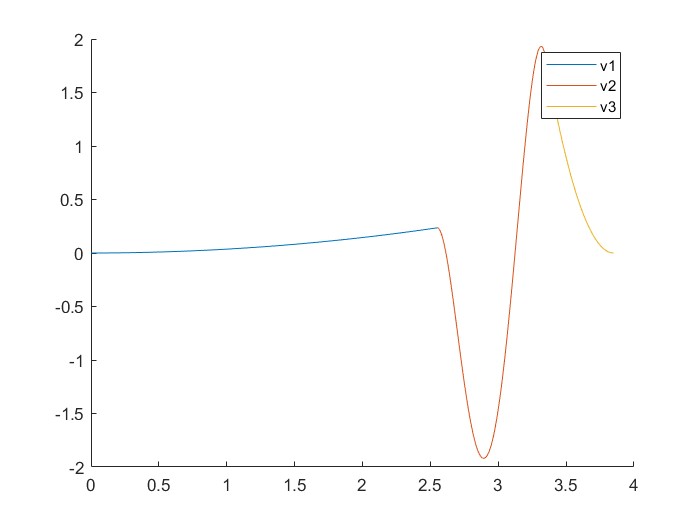

Supplement: Supplementary file 1 — Supplementary Figures. [file 41598_2023_47001_MOESM1_ESM.zip › Supplementary Figures/Figure 14.(d)Simulation Experiment 4.jpg]

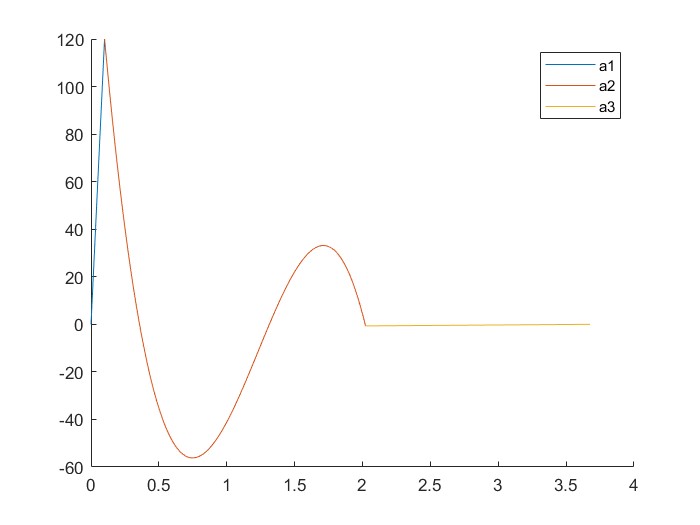

Supplement: Supplementary file 1 — Supplementary Figures. [file 41598_2023_47001_MOESM1_ESM.zip › Supplementary Figures/Figure 15.(a)Simulation Experiment 1.jpg]

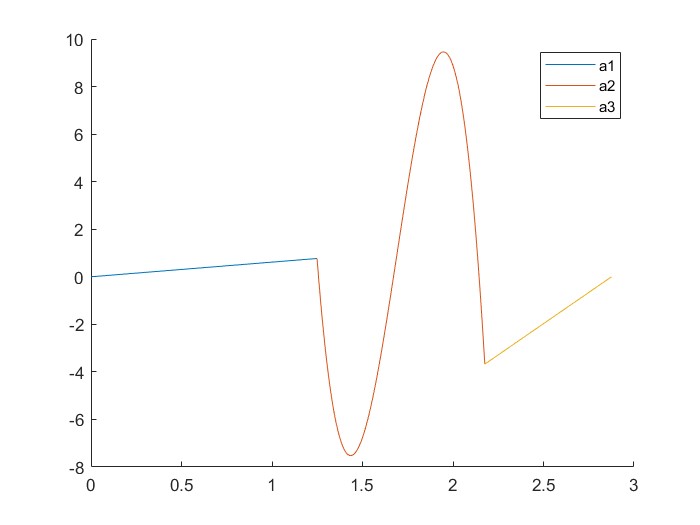

Supplement: Supplementary file 1 — Supplementary Figures. [file 41598_2023_47001_MOESM1_ESM.zip › Supplementary Figures/Figure 15.(b)Simulation Experiment 2.jpg]

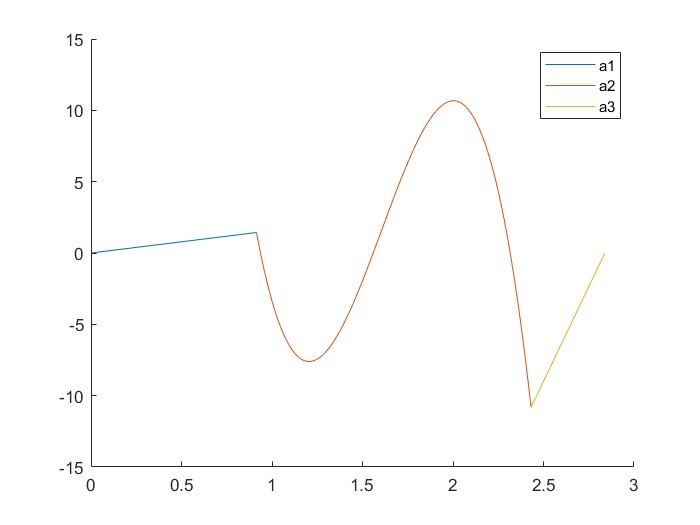

Supplement: Supplementary file 1 — Supplementary Figures. [file 41598_2023_47001_MOESM1_ESM.zip › Supplementary Figures/Figure 15.(c)Simulation Experiment 3.jpg]

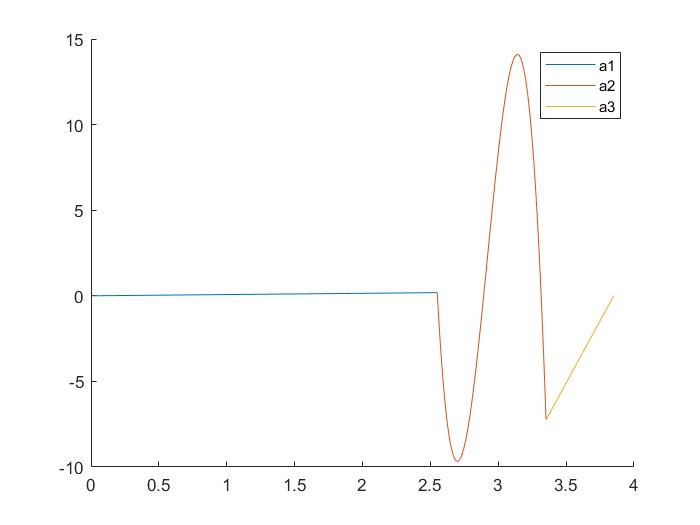

Supplement: Supplementary file 1 — Supplementary Figures. [file 41598_2023_47001_MOESM1_ESM.zip › Supplementary Figures/Figure 15.(d)Simulation Experiment 4.jpg]

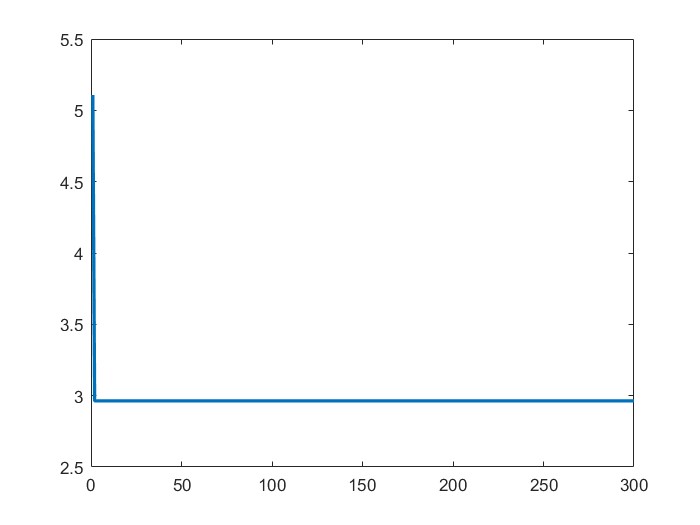

Supplement: Supplementary file 1 — Supplementary Figures. [file 41598_2023_47001_MOESM1_ESM.zip › Supplementary Figures/Figure 16.(a)Simulation Experiment 1.jpg]

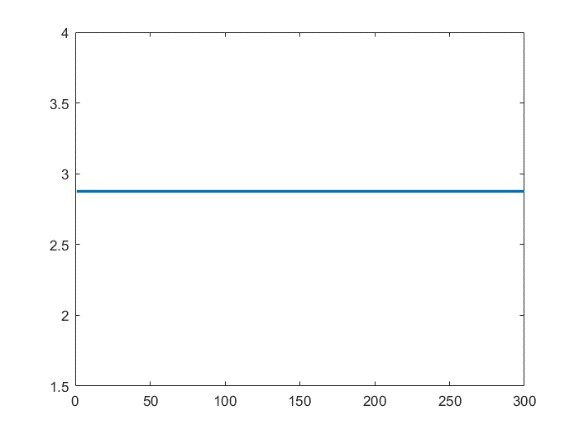

Supplement: Supplementary file 1 — Supplementary Figures. [file 41598_2023_47001_MOESM1_ESM.zip › Supplementary Figures/Figure 16.(b)Simulation Experiment 2.jpg]

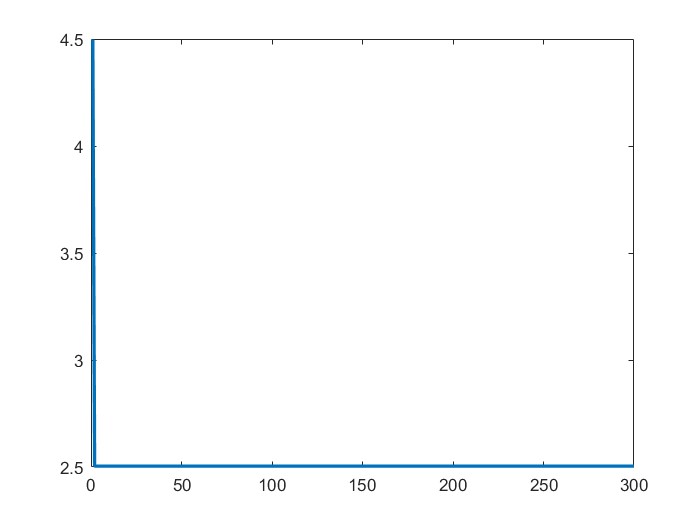

Supplement: Supplementary file 1 — Supplementary Figures. [file 41598_2023_47001_MOESM1_ESM.zip › Supplementary Figures/Figure 16.(c)Simulation Experiment 3.jpg]

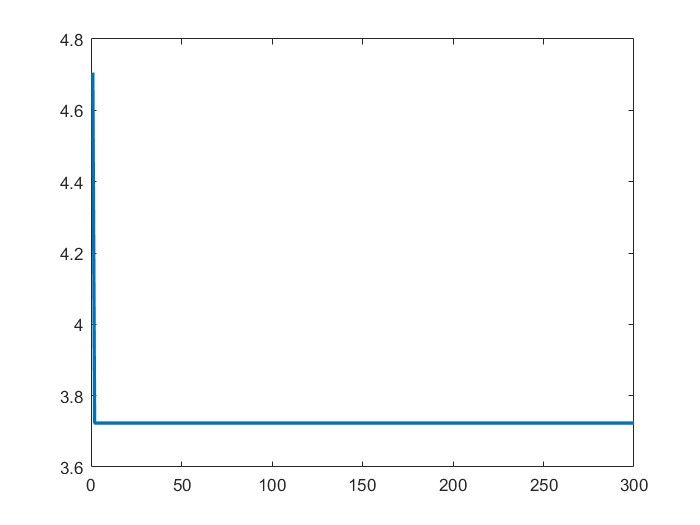

Supplement: Supplementary file 1 — Supplementary Figures. [file 41598_2023_47001_MOESM1_ESM.zip › Supplementary Figures/Figure 16.(d)Simulation Experiment 4.jpg]

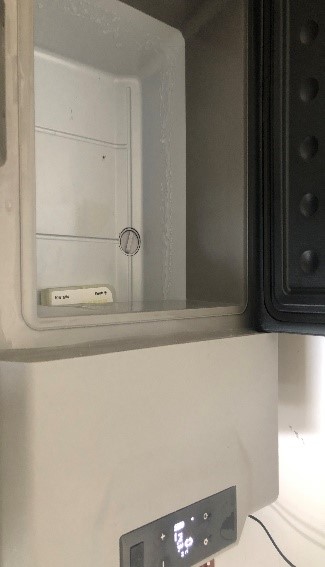

Supplement: Supplementary file 1 — Supplementary Figures. [file 41598_2023_47001_MOESM1_ESM.zip › Supplementary Figures/Figure 17. Refrigeration unit.jpg]

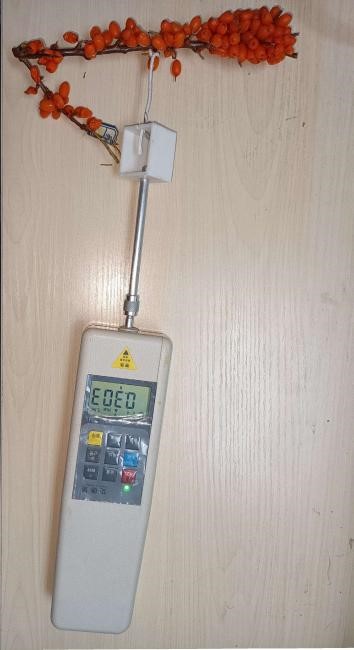

Supplement: Supplementary file 1 — Supplementary Figures. [file 41598_2023_47001_MOESM1_ESM.zip › Supplementary Figures/Figure 18. Force measuring device.jpg]

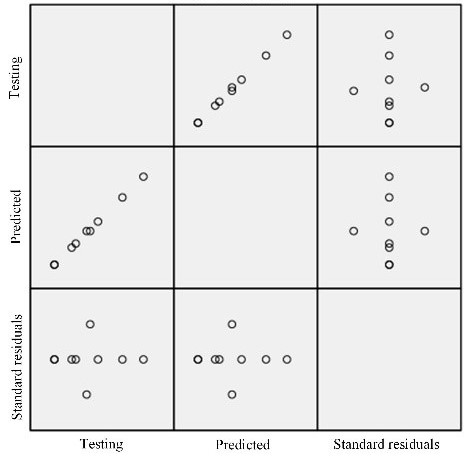

Supplement: Supplementary file 1 — Supplementary Figures. [file 41598_2023_47001_MOESM1_ESM.zip › Supplementary Figures/Figure 19. Standard residual diagram of fruit drop rate.jpg]

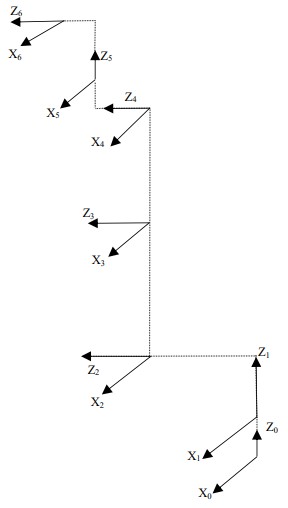

Supplement: Supplementary file 1 — Supplementary Figures. [file 41598_2023_47001_MOESM1_ESM.zip › Supplementary Figures/Figure 2. Coordinates of each joint of the manipulator.jpg]

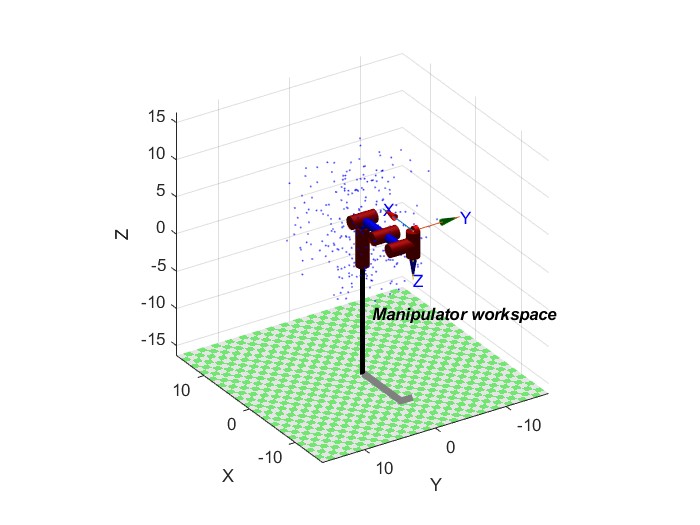

Supplement: Supplementary file 1 — Supplementary Figures. [file 41598_2023_47001_MOESM1_ESM.zip › Supplementary Figures/Figure 3. The 3D model and operating range of the manipulator are established by using the algorithm..jpg]

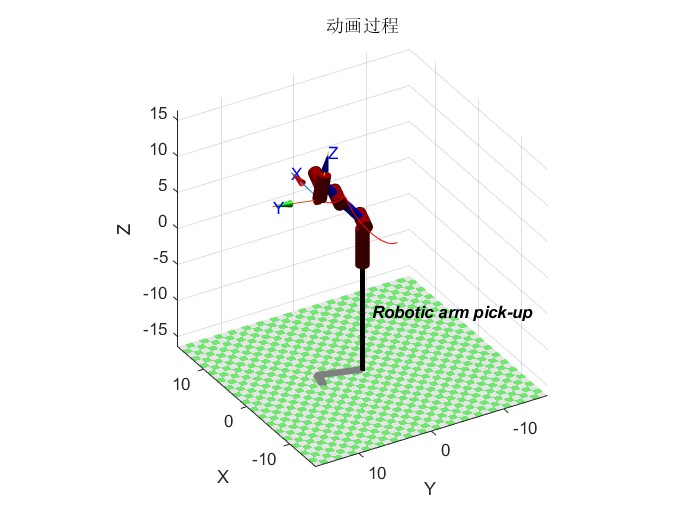

Supplement: Supplementary file 1 — Supplementary Figures. [file 41598_2023_47001_MOESM1_ESM.zip › Supplementary Figures/Figure 4. JTRAJ function planning manipulator to pick up Seabuckthorn branch path..jpg]

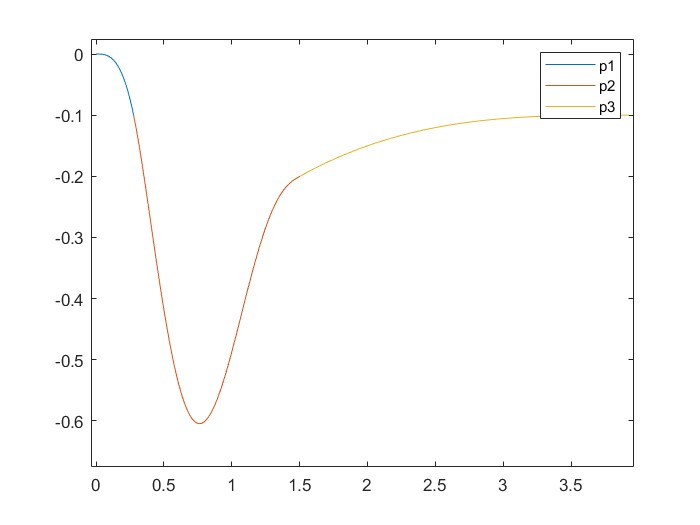

Supplement: Supplementary file 1 — Supplementary Figures. [file 41598_2023_47001_MOESM1_ESM.zip › Supplementary Figures/Figure 5.(a)Simulation Experiment 1.jpg]

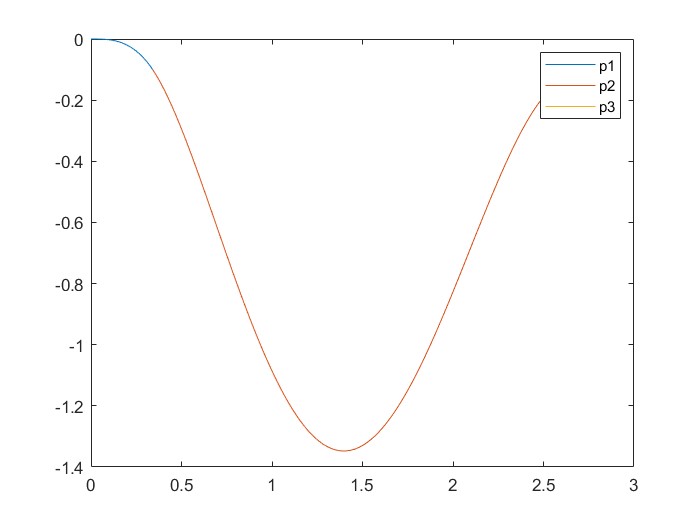

Supplement: Supplementary file 1 — Supplementary Figures. [file 41598_2023_47001_MOESM1_ESM.zip › Supplementary Figures/Figure 5.(b)Simulation Experiment 2.jpg]

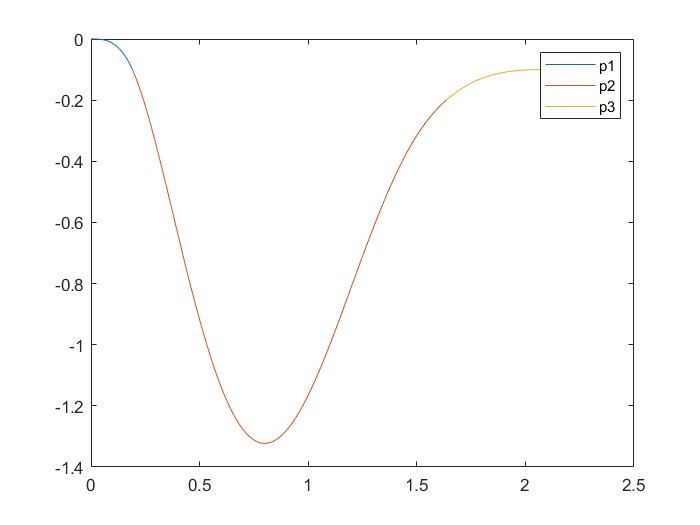

Supplement: Supplementary file 1 — Supplementary Figures. [file 41598_2023_47001_MOESM1_ESM.zip › Supplementary Figures/Figure 5.(c)Simulation Experiment 3.jpg]

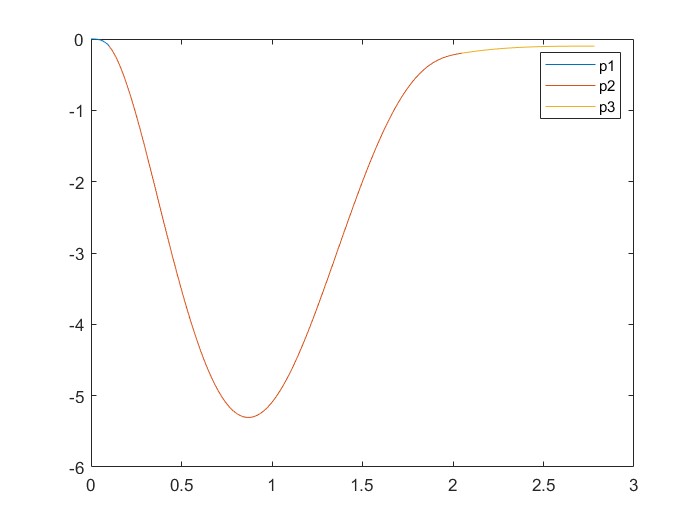

Supplement: Supplementary file 1 — Supplementary Figures. [file 41598_2023_47001_MOESM1_ESM.zip › Supplementary Figures/Figure 5.(d)Simulation Experiment 4.jpg]

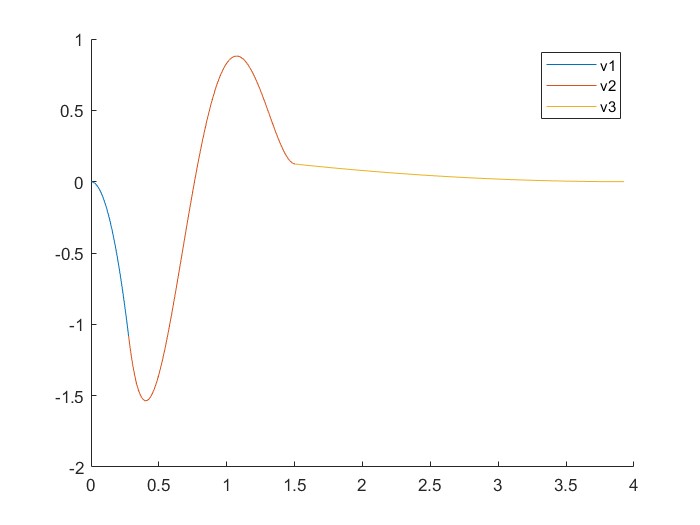

Supplement: Supplementary file 1 — Supplementary Figures. [file 41598_2023_47001_MOESM1_ESM.zip › Supplementary Figures/Figure 6.(a)Simulation Experiment 1.jpg]

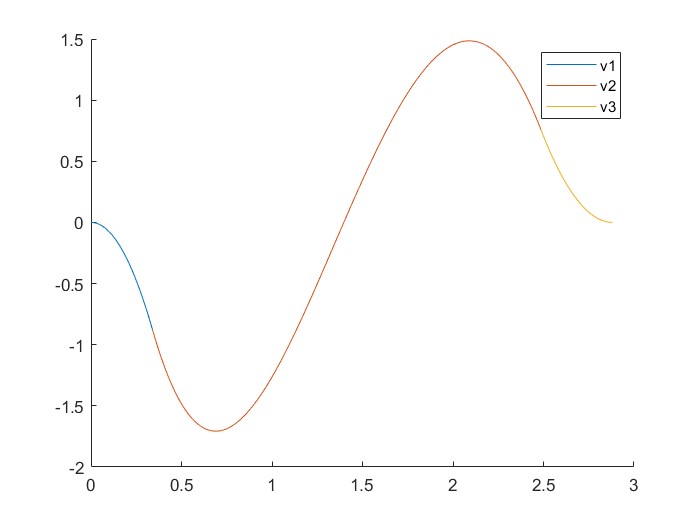

Supplement: Supplementary file 1 — Supplementary Figures. [file 41598_2023_47001_MOESM1_ESM.zip › Supplementary Figures/Figure 6.(b)Simulation Experiment 2.jpg]

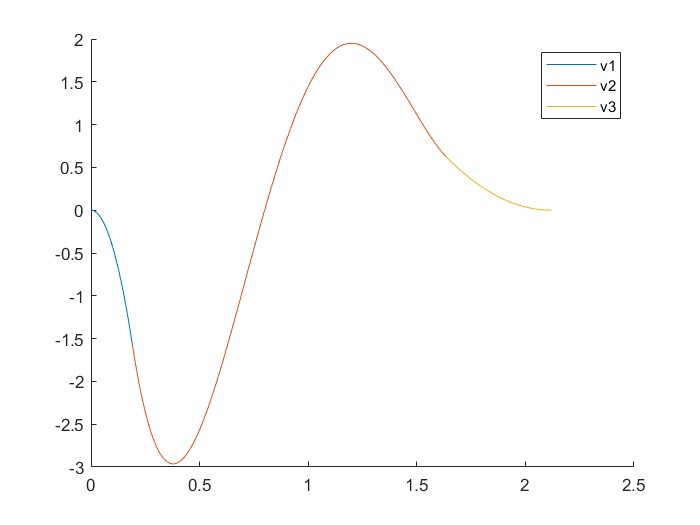

Supplement: Supplementary file 1 — Supplementary Figures. [file 41598_2023_47001_MOESM1_ESM.zip › Supplementary Figures/Figure 6.(c)Simulation Experiment 3.jpg]

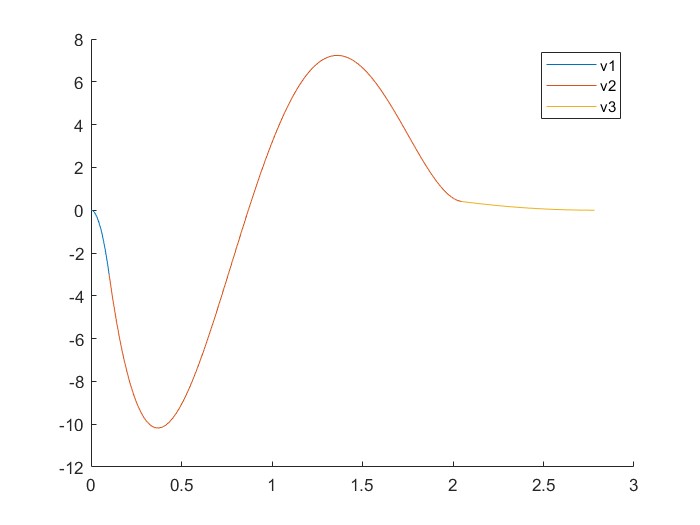

Supplement: Supplementary file 1 — Supplementary Figures. [file 41598_2023_47001_MOESM1_ESM.zip › Supplementary Figures/Figure 6.(d)Simulation Experiment 4.jpg]

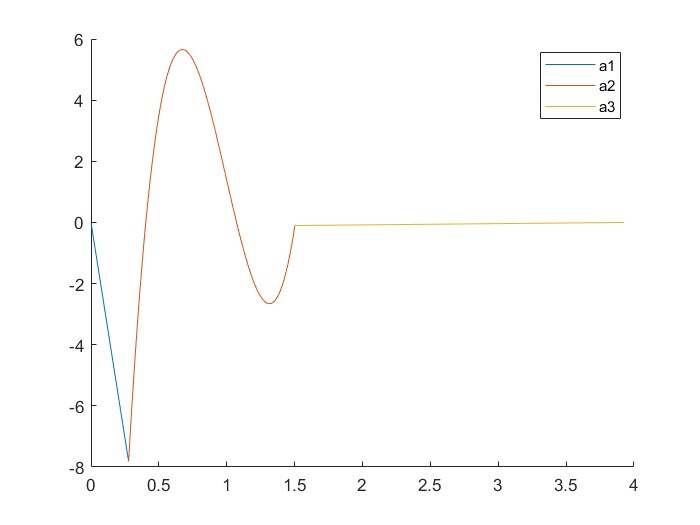

Supplement: Supplementary file 1 — Supplementary Figures. [file 41598_2023_47001_MOESM1_ESM.zip › Supplementary Figures/Figure 7.(a)Simulation Experiment 1.jpg]

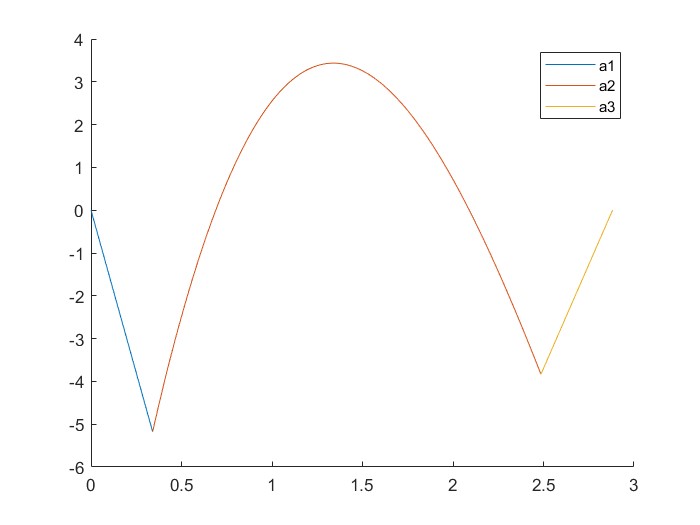

Supplement: Supplementary file 1 — Supplementary Figures. [file 41598_2023_47001_MOESM1_ESM.zip › Supplementary Figures/Figure 7.(b)Simulation Experiment 2.jpg]

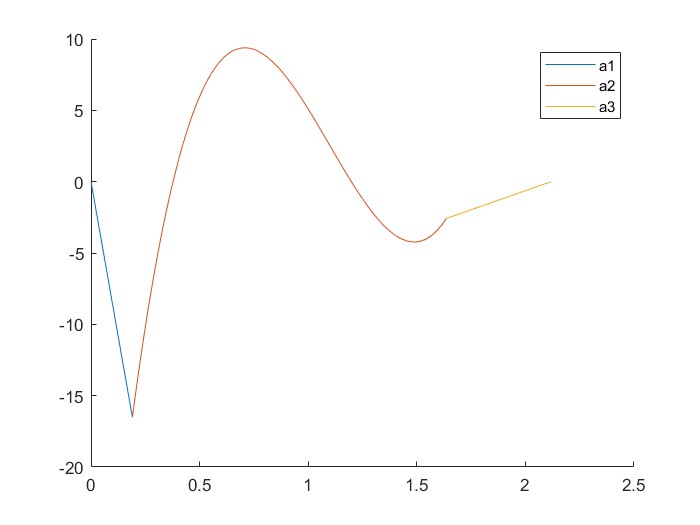

Supplement: Supplementary file 1 — Supplementary Figures. [file 41598_2023_47001_MOESM1_ESM.zip › Supplementary Figures/Figure 7.(c)Simulation Experiment 3.jpg]

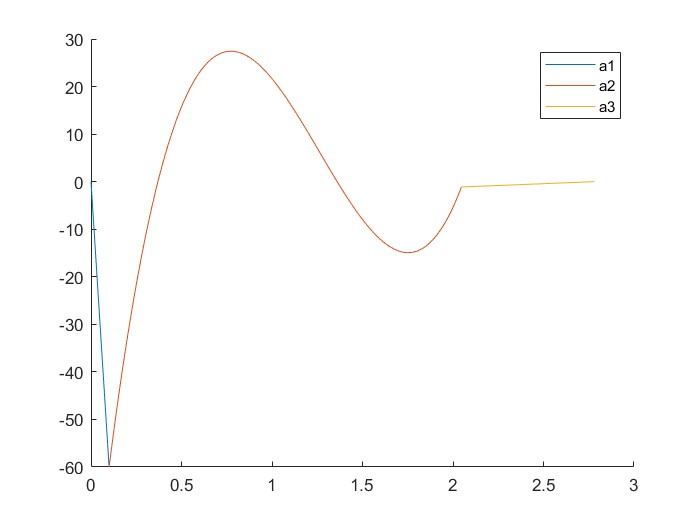

Supplement: Supplementary file 1 — Supplementary Figures. [file 41598_2023_47001_MOESM1_ESM.zip › Supplementary Figures/Figure 7.(d)Simulation Experiment 4.jpg]

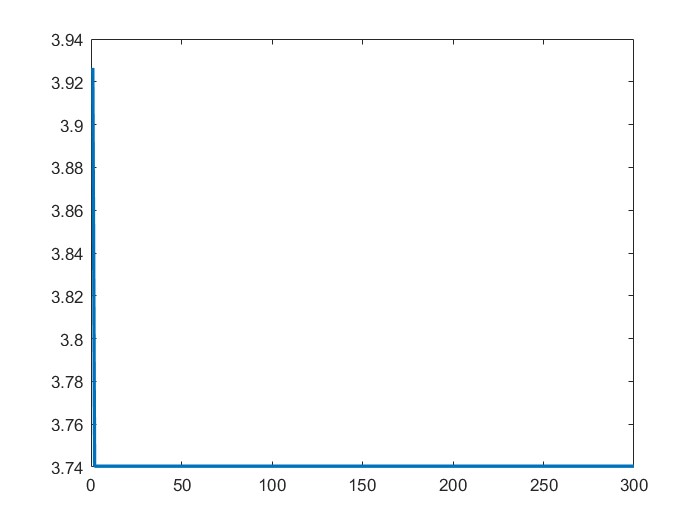

Supplement: Supplementary file 1 — Supplementary Figures. [file 41598_2023_47001_MOESM1_ESM.zip › Supplementary Figures/Figure 8.(a)Simulation Experiment 1.jpg]

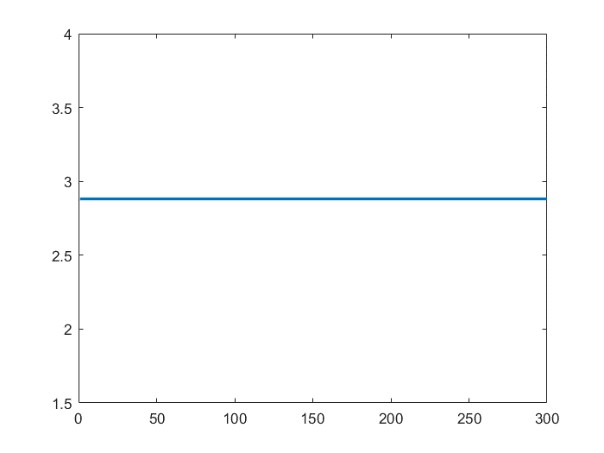

Supplement: Supplementary file 1 — Supplementary Figures. [file 41598_2023_47001_MOESM1_ESM.zip › Supplementary Figures/Figure 8.(b)Simulation Experiment 2.jpg]

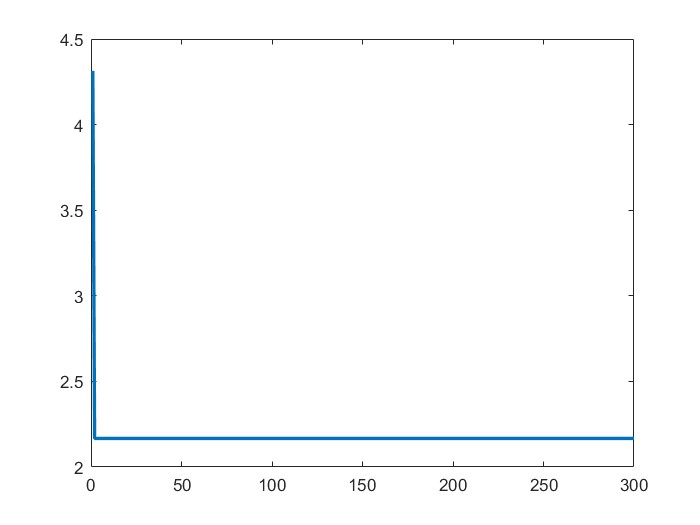

Supplement: Supplementary file 1 — Supplementary Figures. [file 41598_2023_47001_MOESM1_ESM.zip › Supplementary Figures/Figure 8.(c)Simulation Experiment 3.jpg]

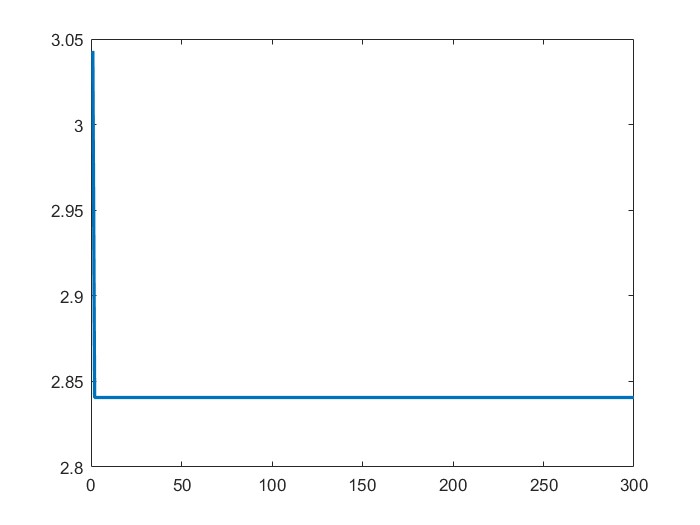

Supplement: Supplementary file 1 — Supplementary Figures. [file 41598_2023_47001_MOESM1_ESM.zip › Supplementary Figures/Figure 8.(d)Simulation Experiment 4.jpg]

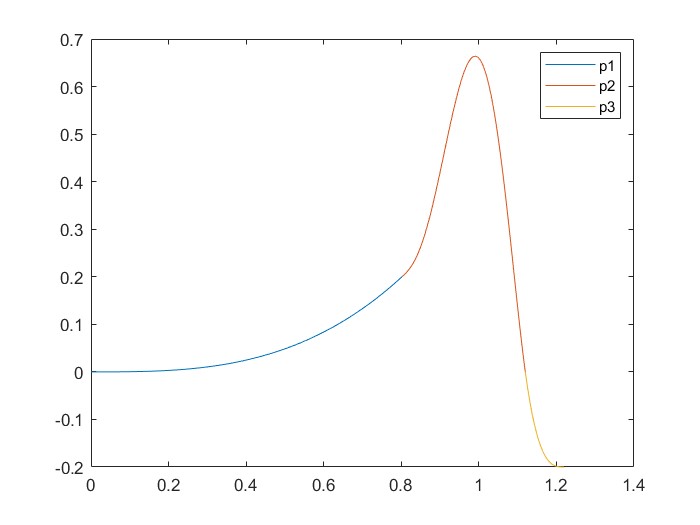

Supplement: Supplementary file 1 — Supplementary Figures. [file 41598_2023_47001_MOESM1_ESM.zip › Supplementary Figures/Figure 9.(a)Simulation Experiment 1.jpg]

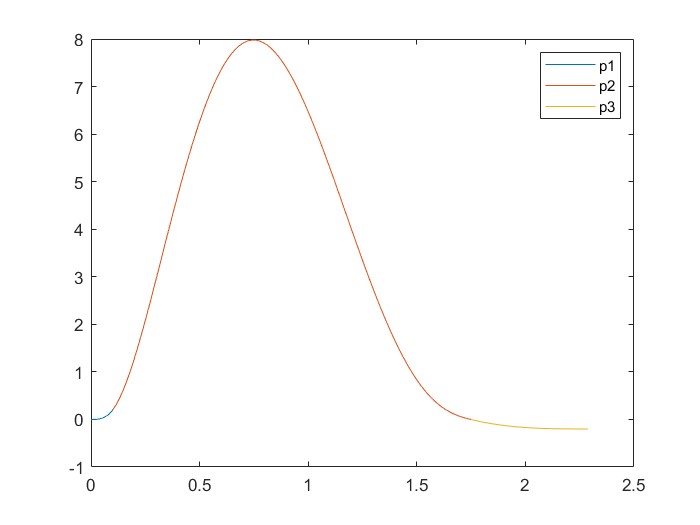

Supplement: Supplementary file 1 — Supplementary Figures. [file 41598_2023_47001_MOESM1_ESM.zip › Supplementary Figures/Figure 9.(b)Simulation Experiment 2.jpg]

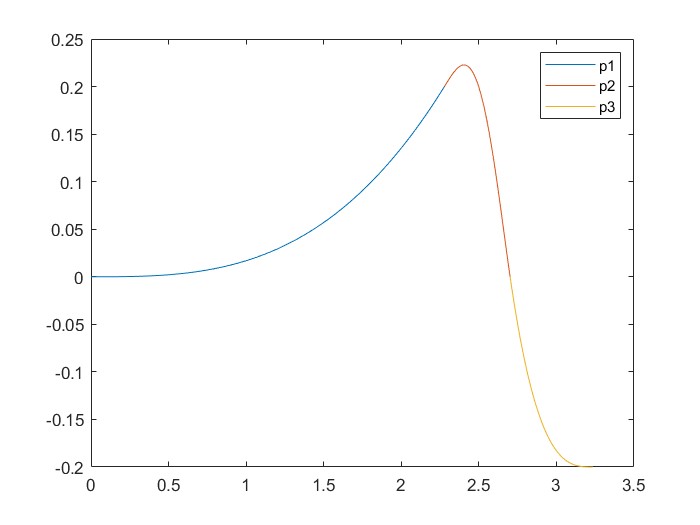

Supplement: Supplementary file 1 — Supplementary Figures. [file 41598_2023_47001_MOESM1_ESM.zip › Supplementary Figures/Figure 9.(c)Simulation Experiment 3.jpg]
